# Immunization timeliness and its determinants among young children in Baghdad, Iraq: A cross-sectional study using documented vaccination cards

**DOI:** 10.1371/journal.pone.0359962

**Published:** 2026-10-01

**Authors:** Syed Shaukat Ali Muttaqi Shah, Hakeem Ahmed Mukhlif Al-ani, Mohammed Abdul Wahab Alwardi, Ale Zehra, Uroosa Kanwal, Tehreem Ansari

**Affiliations:** 1 Department of Clinical Pharmacy, College of Pharmacy, Al-Farahidi University, Baghdad, Iraq; 2 Department of Pharmacognosy, College of Pharmacy, Al-Farahidi University, Baghdad, Iraq; 3 Department of Pharmacy Practice, Faculty of Pharmaceutical Sciences, Dow College of Pharmacy, Dow University of Health Sciences, Karachi, Sindh, Pakistan; 4 Department of Pharmacy Practice, Faculty of Pharmacy, Salim Habib University, Karachi, Sindh, Pakistan; 5 Department of Infectious Diseases, Dow University Hospital (Ojha Campus), Dow University of Health Sciences, Karachi, Sindh, Pakistan; PLOS: Public Library of Science, ETHIOPIA

## Abstract

**Background:**

Childhood immunization is a critical public health intervention, yet timeliness remains a challenge in post conflict affected settings like Iraq. This study assessed timeliness of immunization, missed doses, and associated factors with delay among children aged 18–24 months in Baghdad, Iraq.

**Methods:**

A cross-sectional study was conducted in 11 primary healthcare centers in Baghdad between May and July 2025. Data were collected from 168 children using documented immunization cards. Immunization delay was defined as vaccination administered beyond >28 days of the scheduled date. Multivariate logistic regression was used to identify associated factors.

**Results:**

Overall vaccine coverage (timely + delayed) remained high (>96%) for first nine months, at 12 and 18 months, coverage declined to 85.1% and 63.7%, respectively. Only 14 children (8.3%) received all vaccinations on time. Immunization delay was observed in 154 (91.7%) children, with 28 (16.7%) of children with delay at a single visit and 126 (75.0%) at multiple visits. Missed doses were highest at 18 months (36.3%), followed by 12 months (14.9%). Timely vaccination dropped sharply from 98.6% at birth to 52.4% at 9 months, and 37.5% at 18months. The most common reason for delay was lack of knowledge about the vaccination schedule (54.8%). In adjusted analysis, lack of knowledge of schedule (aOR = 3.85; 95% CI: 1.04–14.24) and perceived vaccine availability (aOR = 4.74; 95% CI: 1.09–20.51) were significantly associated with delay. No sociodemographic factors were significant.

**Conclusion:**

Immunization delay is highly prevalent among children in Baghdad. Interventions focusing on caregiver knowledge and ensuring consistent vaccine supply are urgently needed, particularly for children in the second year of life. However, due to purposive sampling, these findings may not be generalizable to all of Baghdad or Iraq.

## Introduction

Vaccination is one of the most important public health interventions in the history of medicine, providing primary prevention of serious infections [1]. Globally, vaccinations are estimated to prevent around 2–3 million deaths every year [2]. Childhood vaccination is the cornerstone of public health interventions, playing a vital role in preventing infectious diseases, and significantly reducing morbidity and mortality rates [1,3]. The World Health Organization (WHO) established the expanded program for immunization (EPI), which has significantly reduced vaccine-preventable diseases and deaths since 1974 [4]. EPI aims to extend new vaccines and preventive health interventions to all the parts of the world, originally the program was focused on six diseases (tuberculosis, diphtheria, pertussis, polio and measles), it has expanded to include 13 universally recommended vaccines, including Covid-19 [5]. These vaccination programs have contributed vitally to the eradication of diseases such as polio, small-pox, measles and tetanus globally [6].

Compliance to childhood vaccination is challenging; globally immunization programs reach over 80% of children under one year of age, preventing around 2.5 million deaths per year, while it is estimated if the coverage increases to 90% it can prevent 2 million more deaths annually [7]. In order to increase the coverage and compliance, a number of actions like scientific advancement in production, simplified delivery methods, uninterrupted supplies of vaccination, and social/political commitment to the vaccination program is highly warranted [8]. On-time vaccination is important to protect children from vaccine preventable diseases, and children who receive vaccination later than recommended schedule remain susceptible to these diseases [9]. Delay in immunization has serious consequences on health systems especially during an outbreak [10].

Iraq had one of the most advanced healthcare systems in the Middle East, achieving universal health from 1960s to 1980s [11]. However, healthcare system has been significantly impacted by the decades of conflict and instability [12]. Despite improvements in the immunization system over the past decade, the country is still struggling to provide the vaccines to all the children, making vaccine-preventable diseases as a primary cause of child morbidity and mortality [7]. According to a recent report from the Iraqi Ministry of Health, the Polio, BCG and Measles vaccination rates are above 90%; however 1.5 million children still need to be fully vaccinated, indicating a significant gap between the immunization requirements and actual compliance [13]. Amongst common factors for the low compliance regarding childhood immunization in Iraq, inadequate parental awareness has been identified as one of the major reason [14]. Misconceptions related to safety and efficacy of vaccines, religious beliefs, and lack of trust in government policies also results in noticeable vaccine hesitancy and refusal [6,15].

This study was conducted in Baghdad, Iraq to assess compliance with the childhood vaccination focusing on children aged 18–24 months, as this age group should have completed all seven essential immunization visits recommended by the Iraqi Ministry of Health. The specific aims of the study were to: (1) determine the proportion of children with timely, delayed (single or multiple visits), and missed vaccinations across all seven essential immunization visits (1^st^ week, 2, 4, 6, 9, 12, and 18 months); (2) examine the association of delayed immunization with caregiver knowledge, beliefs, education, socioeconomic status, and family size; and (3) identify reasons for immunization delay.

This study is novel in several aspects. First, it assesses immunization timeliness across all seven essential visits using documented immunization cards rather than maternal recall. Second, it is the first study in Iraq to provide a characterization of non-compliance patterns, distinguished between single or multiple visit delays and missed doses of vaccination. Third, it evaluates the impact of both vaccine availability and caregiver knowledge on immunization delay for children under two years of age in a post-conflict urban setting.

## Methodology

### Study design, setting, and duration

An observational cross-sectional study was conducted in 11 primary healthcare centres (PHCs) of Baghdad, Iraq, between May 8^th^ 2025 and July 31^st^ 2025. Baghdad represents a post-conflict healthcare context due to decades of war and instability, experienced multiple conflicts since 1980, including Iran-Iraq war (1980–1988), the Gulf war (1990–1991), 2003 invasion and occupation, and the ISIS conflict (2014–2017). Although major active conflict subsided for more than two decades, the healthcare system continues to struggle with cumulative effects. Availability of healthcare worker is still a challenge with approximately 1.8 physicians per 1000 population, lower than numbers of 2003 [11,12]. Many primary health care centres face resource challenges, and the cold-chain compromised by electricity shortages [12]. This post-conflict context pose unique challenges for vaccination program, making Baghdad an important setting for evaluating immunization timeliness [13].

Baghdad has a mix of public and private health care facilities. Primary Healthcare Centres (PHCs) are the main public health facility, providing free of charge preventive and curative services, that include routine immunization. PHCs in urban Baghdad normally serve to populations of 15,000–50,000 residents in the neighbourhoods, as the first point of contact for maternal and child health services. These PHCs deliver EPI services, which make them strategically important for the evaluation of vaccination program performance in the urban setting [11,13]. Furthermore, Baghdad is the administrative capital of Iraq and is the most densely populated urban community in Iraq; conducting this study in the city makes it a critical site for evaluating national health initiatives like vaccination programs and can provide insight into challenges within a major metropolitan setting.

### Study population and their selection criteria

The primary purpose of this study was to examine the immunization timeliness among the young children of Baghdad city, Iraq. This study chiefly targeted young children aged between 18 and 24 months, because by the age of 18 months, a child should have received basic immunization proposed by the Iraqi Ministry of Health [16], by the age of 18 months, a child should have made seven essential immunization visits, completing both primary doses (birth till 12-month) and booster doses at 18-month [17]. These seven immunization visits should be carried out at childbirth, second month, fourth month, sixth month, ninth month, 12 months and 18 months of their life. These seven essential immunization visits included: birth (1st week: BCG, OPV-0, HepB-1), 2 months (DTaP-HepB-Hib-1, OPV-1, PCV-1, Rotavirus-1), 4 months (DTaP-HepB-Hib-2, OPV-2, PCV-2, Rotavirus-2), 6 months (DTaP-HepB-Hib-3, OPV-3, PCV-3), 9 months (Measles-1), 12 months (MMR-1, Varicella-1), and 18 months (DTaP-IPV-Hib Booster, MMR-2, Varicella-2) [16,17]. One of the basic criteria of this study participation was to provide documented evidence regarding the child’s age and child immunization, the age window (18–24 months) was selected to minimize the recall bias as immunization cards are still typically retained by caregivers during this period. Children who failed to present immunization card at the time of data collection were excluded from the study participation irrespective of their physical and biological age. Furthermore, any child who had not reached the age of 18 months at the time of data collection was excluded from the study, because their inclusion may have either overestimated or underestimated immunization compliance at any immunization visit. Therefore, this study only considered those children who were eligible to reach the age of seven essential immunization visits, i.e., children aged over 18 months.

### Sample size

Sample size was calculated by taking immunization coverage into consideration from previous study in Iraq, that reported 83.7% of measles vaccination [18]. Using OpenEPI software with a 95% confidence interval, a 5.6% margin of error, the minimum required sample was calculated as 168. The calculated sample size provides 80% power to detect a 20% difference in immunization delay at 5% significance level. Furthermore, the sample size exceeded the minimum requirement of 10 event per predictor variable for logistic regression analysis. Therefore, a target sample of 168 participants was considered adequate.

### Sampling method

While random sampling was not feasible due to logistic constraints and need of prior administrative approval of primary healthcare centres (PHCs), a multistage stratified purposive sampling technique was used.

Stage 1 – Selection of PHCs: Thirty PHCs were contacted across Baghdad’s two main sectors (Kurkh and Rusafa). PHCs were purposively selected based on their willingness to grant permission for data collection after formal application for data collection, resulting in 11 participating PHCs out of 30 contacted.

Stage 2 – Selection of participants: Within each selected PHC, caretakers were purposively approached based on the convenience and availability at the time of data collection. Data collectors approached caregivers attending the PHC for any reason (immunization, curative care, or other services) and screened them for eligibility (children aged 18–24 months with immunization cards)

Stage 3 – Enrolment: Eligible caregivers were invited for the willingness to participate, provided written consent and their children’s immunization cards were used for data collection.

This approach was used because PHCs approval was required, and only centres granting permission could be included; the study required access to vaccination cards and probability of getting them was higher during a PHC visit; and stratification by sector ensured geographic diversity.

### Data collection tool

In this study, the data was collected using a pre-validated and structured questionnaire, which was adopted from Khaliq et al. (2017) [19] and is provided as “S1 File”. The questionnaire used in this study consist of 63 questions, which were spread into seven different sections. Each section has different components. The first three sections of the questionnaire were related to the participant’s screening, eligibility, consent and enrolment, while the last four sections asked questions related to participant demography, participant knowledge and behaviour, participant immunization status at each immunization visit, collected vaccination dates from cards, and perceived reason of immunization delay.

### Study variables

The immunization status of children aged at 18 months or over was assessed by inspecting their immunization card. Following immunization card assessments, we derived two different types of immunizations outcomes: valid immunization and delayed immunization.

Immunization delay was defined as vaccination administered beyond 28 days of the scheduled date. This 4-week window was selected based on: WHO recommendations for immunization timeliness assessment, allowing a grace period for scheduling variations while maintaining protective immunity; and, consistency with published literature from similar settings that have used 28 days window period to define vaccination timeliness [6,19,20]. The Iraqi Ministry of Health provides the recommended age for vaccination [16], but does not explicitly define an acceptable delay window; therefore 28 days definition was adopted from the literature [6,19,20]. Children who received vaccinations within the 28-day window were classified as valid immunization, as this timeframe is considered clinically acceptable for maintaining age-appropriate protection, while any children who received immunization beyond 28 days window periods were categorized to have *delayed immunization*. The delayed immunization was of two types – *immunization delayed at a single visit and immunization delayed at multiple visits*. A *missed vaccination* was defined as a vaccine that was not administered by the time of data collection (i.e., no record of vaccination on the card for a particular scheduled visit by 18–24 months of age). This is distinct from *delayed vaccination*, where the vaccine was administered but beyond the 28-day window. Apart from immunization timing, various maternal, paternal, household and child factors were also taken into consideration.

The maternal factors include maternal age (less than 20 years, 20–29 years, 30–39 years and 40–49 years) and maternal education (no education, primary, secondary, and higher). Similarly, the *paternal factors:* The paternal factors include paternal age (less than 20 years, 20–29 years, 30–39 years and 40–49 years) and paternal education (no education, primary, secondary, and higher). However, child sex (male and female) and birth order (first birth and subsequent birth) were considered as child factors. The household factors comprise of family size (small family and medium to large family) and type of place of delivery (home delivery and institutional delivery). Households having family size of five or less than five members were classified as small family sized households, whereas those with over five family members were grouped into medium to large sized households. Similarly, the place of delivery was also bifurcated into two major groups: institutional delivery and home delivery. Children born in any public and private clinic or hospital or maternity centre or basic health units were grouped as institutional delivery, while those born in their own or their relative or neighbour or friends house were classified to have home delivery. The data dictionary defining all variables and coding is available as “S2 File”.

### Data collection procedure and management

The data of this study was collected by a team of pharmacy final year students studying at Al-Farahidi University. All the students received a formal training of two weeks from their supervisor. The training included how to approach potential participants, describing the study purpose, pre-enrolment screening, eligibility determination, consent taking procedure, and enrolment. Moreover, all the data collectors also received soft skills training for data collection, such as how to approach, how to greet, and how to conclude the interview. The project supervisor divided different tasks to a groups of data collector each week, which include data collection, data entry, and data quality assurance (spot-checks and double data entry) for reducing the unintentional missing data and for improving the quality and transparency of the collected data.

### Data analysis

The data of this study was analysed at three different steps. At first, preliminary analysis was performed to examine the distribution of each categorical and numerical variables. Secondly, descriptive statistics were performed in which we calculated the percentages of all the categorical variables, and finally inferential statistics were performed using bivariate logistic regression. Children who received immunization on time were considered as reference, while those who had delayed immunization were tested against the reference. A confidence interval of 95% and a p-value of ≤0.05 was considered as significant value for assessing the association of delay immunization with different covariates. For multivariate logistic regression, variable with p-value ≤0.20 or less in bivariate analysis were considered for model entry. A backward elimination method was adopted, with variables having p-value > 0.05 removed sequentially until the final model contains variables with p-value ≤0.05. The final model included 7 variables: knowledge of vaccination schedule, vaccine availability, and vaccine availability, along with vaccination timeliness at 1, 2, 4, 6, 9, 12, and 18 months. With 154 children experiencing delay (the outcome event) and 7 variables in the final model, the events-per-variable ratio was 22:1, exceeding the recommended minimum of 10:1 for logistic regression analysis. For demographic and refusal reason variables, missing responses were recoded as 0 (indicating absence of the barrier). Vaccination status variables, only children with complete immunization card data were included in the analysis, as incomplete cards were an exclusion criterion. No imputation methods were applied due to the low proportion of missing data (<5% for all variables)

### Ethical considerations

This study was approved by the Research Ethics Committee, Al-Farahidi University, college of Pharmacy, approval number Farahidi-AD no: 1–652025. Furthermore, a formal permission from each primary healthcare centre was also taken prior to the study data collection. A written consent from parents/guardian of the child was obtained, and a complete information about the study purpose, and participant’s rights was provided, including data confidentiality, anonymity, and rights to refuse or withdraw from participation. In addition, risk and benefits associated with the study participation was also provided to each participant before their participation and enrolment. The STROBE checklist for cross-sectional studies is provided as “S3 File”.

The overall study methodology and participant selection process are illustrated in Fig 1

**Fig 1:**
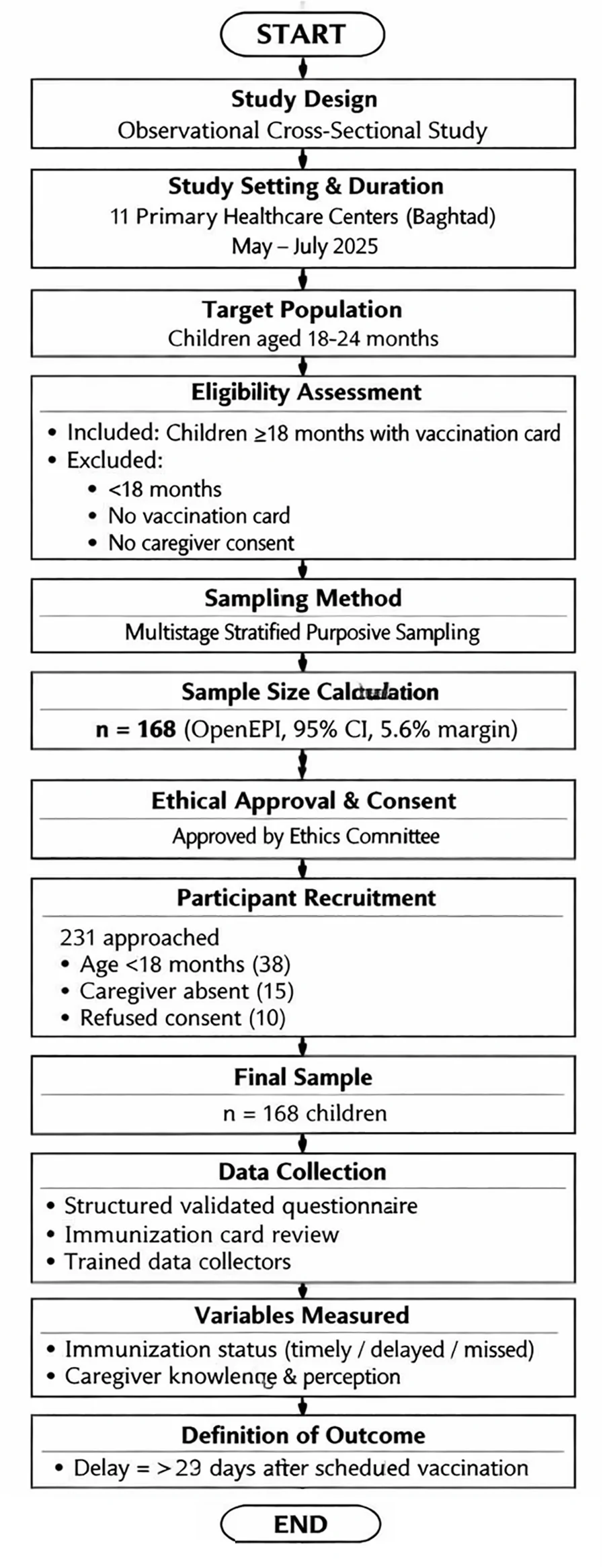
Study flowchart of participant selection and methodology. The figure outlines the sequential steps from initial approach to final inclusion: screening of 231 children, application of eligibility criteria (age 18–24 months, immunization card availability, caregiver presence, and consent), and enrolment of 168 participants into the final analysis.

## Results

### Descriptive analysis of study participants

A total of 231 participants were approached, of these, 63 were excluded from the final analysis. The reasons included: 38 children were below 18 months of age; 15 children excluded because primary caregiver was not present at the time of data collection; and 10 caregivers did not provide consent for study participation, resulting in a final sample size of 168 participants included in this study.

The demographic characteristics of the study participants are presented in Table 1. Among these 168 participants, more than half of mothers were aged below 30 years (62.5%) followed by 30–39 years (34.5%), while less than 5% mothers were of aged between 40–49 years and below 20 years, with the mean (±SD) age 28.4 ± 5.7 years. Regarding maternal education, 6.5% had no formal education, 23.2% had primary education, 50% had secondary education, and 20.2% had higher education. Among fathers, 56% were aged 30–39 years, 33.9% were 20–29 years, and 10.1% were 40–49 years. None were reported to be below 20 years. In terms of paternal education, 4.2% had no education, 25% had primary education, 48.2% had secondary education, and 22.6% had higher education.

**Table 1:** Demographic characteristics of the study sample.

Variables	Categories	N	Percentage
Mothers Age	Between 30 years	105	62.50%
	Between 30–39 years	58	34.50%
	Between 40–49 years	5	3%
Mothers Education	No education	11	6.50%
	Primary	39	23.20%
	Secondary	84	50%
	Higher	34	20.20%
Fathers Age	Between 20–29 years	57	33.90%
	Between 30–39 years	93	55.40%
	Between 40–49 years	17	10.10%
Fathers Education	No education	7	4.20%
	Primary	42	25%
	Secondary	81	48.20%
	Higher	38	22.60%
Gender	Male	92	54.80%
	Female	76	45.20%
Birth order	Index child	62	36.90%
	Subsequent child	106	63.10%
Place of birth	Home delivery	9	5.40%
	Institutional delivery	159	94.60%
Family size	Small	81	48.20%
	Medium OR large	87	51.80%

Note: Data are presented as frequencies (N) and column percentages. Percentages may not sum to 100% due to rounding. CI = Confidence Interval. SD = Standard Deviation. Categories with small numbers (<5) were merged where appropriate to ensure stable estimates.

Of the children, 54.8% were male and 45.2% were female. Regarding birth order, 36.9% were index children and 63.1% were subsequent children. Most children were born in health institutions (94.6%), while 5.4% were delivered at home. In terms of family size, 48.2% of households were categorized as small, and 51.8% as medium or large.

### Immunization timeliness and coverage

Assessment of vaccination cards revealed that 154 children (91.7%; 95% CI: 86.5–95.2) experienced delayed vaccination at least at one or multiple occasions. Out of these, 28 children (16.7%) were delayed at only one visit (single visit delay), while 126 children (75.0%) experienced delays at two or more visits (multiple visit delay). These categories are mutually exclusive. The remaining 14 children (8.3%) received all scheduled vaccinations within the recommended timeframes or permissible 28 days window, elaborated in Fig 2.

**Fig 2:**
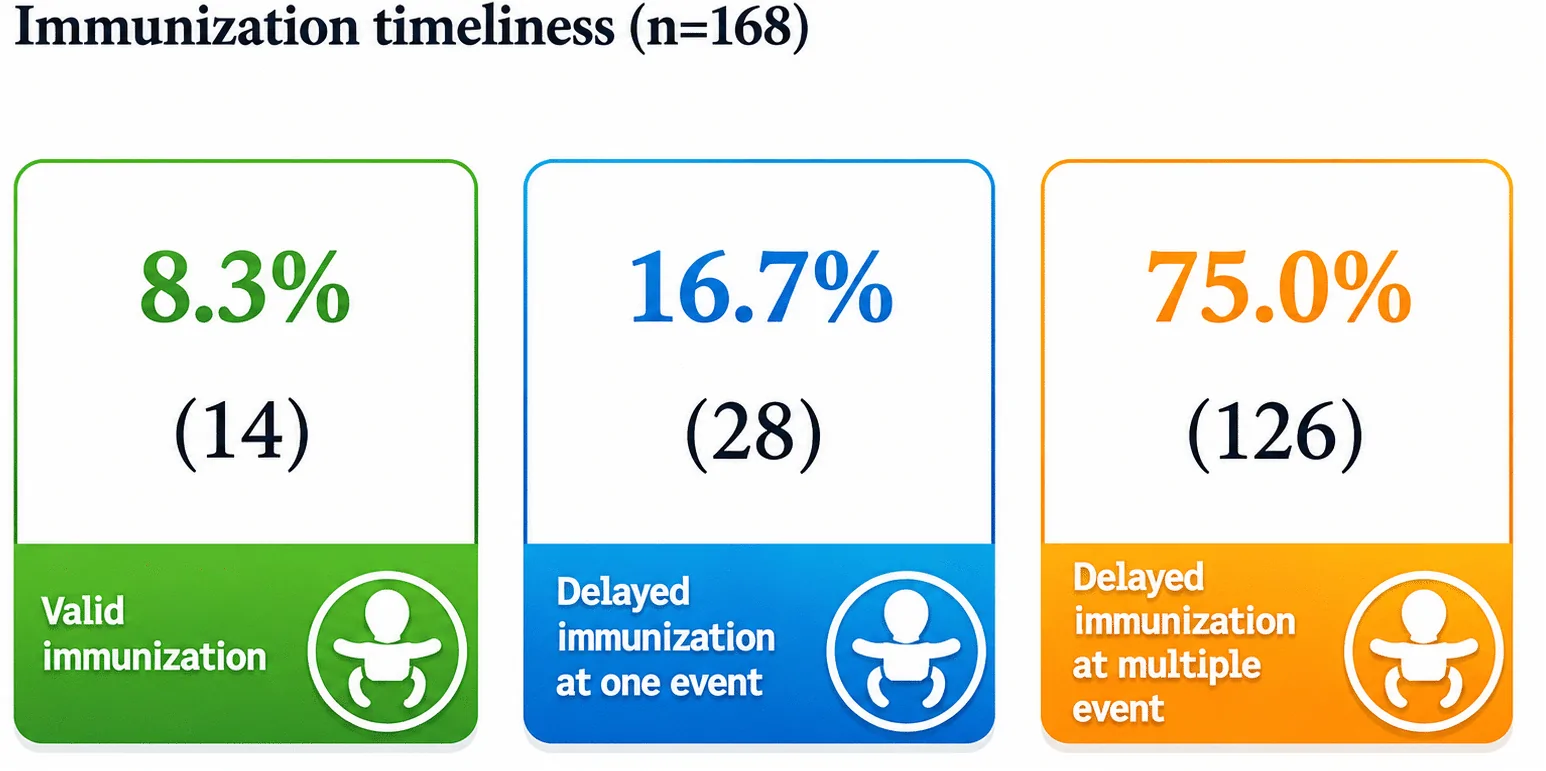
Immunization timeliness (n = 168). Only 14 children (8.3%) received all scheduled vaccinations within the recommended 28-day window. A total of 154 children (91.7%) experienced delays. Among these, 28 children (16.7%) were delayed at only one visit (single visit delay), and 126 children (75.0%) were delayed at two or more visits (multiple visit delay). These categories are mutually exclusive.

### Age-proportionate immunization status

The proportion of children with timely, delayed, and missed vaccinations across seven visits is elaborated in Fig 3. At birth 165 (98.6% 95% CI: 95.6–99.7) received timely vaccination and 3 (1.8%, 95% CI: 0.6–5.1) received delayed vaccination. At the first-week visit, 138 children (82.1%; 95% CI: 75.6–87.5) received timely vaccination, 30 children (17.9%; 95% CI: 12.6–24.4) experienced delays. At the 2-month visit, 124 children (73.8%; 95% CI: 66.6–80.1) received timely vaccination, 43 children (25.6%; 95% CI: 19.4–32.7) experienced delays and 1 (0.6%, 95% CI: 0.1–3.3) child missed the doses. At 4-month visit, 114 children (67.9%; 95% CI: 60.5–74.6) received vaccination timely, 52 children (31.0%; 95% CI: 24.2–38.4) received delayed and 2 (1.2% 95% CI: 0.3–4.2) children missed the doses. At the 6-month visit, 113 children (67.3%; 95% CI: 59.9–74.1) were timely, 54 children (32.1%; 95% CI: 25.3–39.6) delayed and 1 (0.6%, 95%: 0.1–3.3) child missed the doses. At the 9-month visit, 88 children (52.4%; 95% CI: 44.8–59.9) received timely vaccination, 74 children (44.0%; 95% CI: 36.6–51.7) were delayed, and 63 children (3.6%; 95% CI: 1.5–7.6) missed the dose. At the 12-month visit, 100 children (59.5%; 95% CI: 51.9–66.8) were timely, 43 children (25.6%; 95% CI: 19.4–32.7) delayed, and 25 children (14.9%; 95% CI: 10.1–21.0) missed. At the 18-month visit, 63 children (37.5%; 95% CI: 30.4–45.0) received timely vaccination, 44 children (26.2%; 95% CI: 19.9–33.4) were delayed, and 61 children (36.3%; 95% CI: 29.2–43.9) missed the dose.

**Fig 3:**
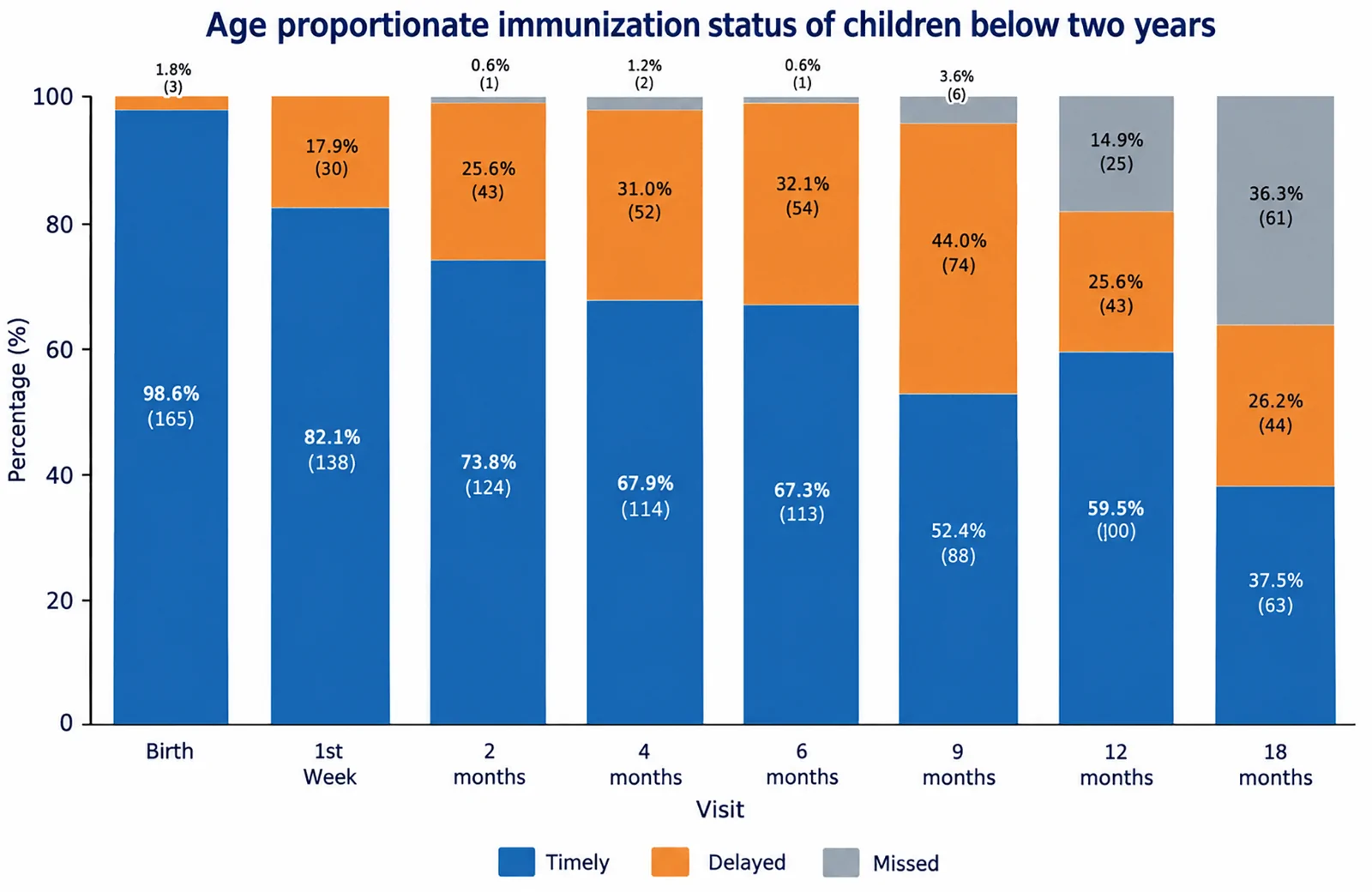
Age proportionate immunization status of children below two years. The figure shows the proportion of children with timely, delayed, and missed vaccinations at each scheduled visit (birth, 2, 4, 6, 9, 12, and 18 months). Error bars represent 95% confidence intervals. Timely vaccination declined sharply after birth, from 98.6% at birth to 52.4% at 9 months and 37.5% at 18 months. Missed doses were highest at 18 months (36.3%), followed by 12 months (14.9%). Valid (Timely) = vaccine administered within 28 days of scheduled date, Delayed = vaccine administered >28 days after scheduled date, Missed = no record of vaccination on card by 18-24 months of age.

### Reasons for immunization delay

Reasons for delayed immunization as per the caregivers are presented in Fig 4. The most frequently mentioned reason of delayed vaccination was lack of knowledge regarding the vaccination schedule 92 (54.8%; 95% CI: 47.1–62.3). Other reasons included family against vaccination 32 (19.0%; 95% CI: 13.6–25.8), negative perception about vaccination 30 (17.9%; 95% CI: 12.6–24.5), vaccine price 27 (16.1%; 95% CI: 11.1–22.4), and vaccine-related adverse drug reactions (ADRs) 27 (16.1%; 95% CI: 11.1–22.4). Child trauma was reported by 22 caregivers (13.1%; 95% CI: 8.6–19.1), vaccine availability issues by 21 (12.5%; 95% CI: 8.1–18.4), child illness by 16 (9.5%; 95% CI: 5.7–15.0), and family migration was the least frequently cited reason, reported by only 6 caregivers (3.6%; 95% CI: 1.5–7.7).

**Fig 4:**
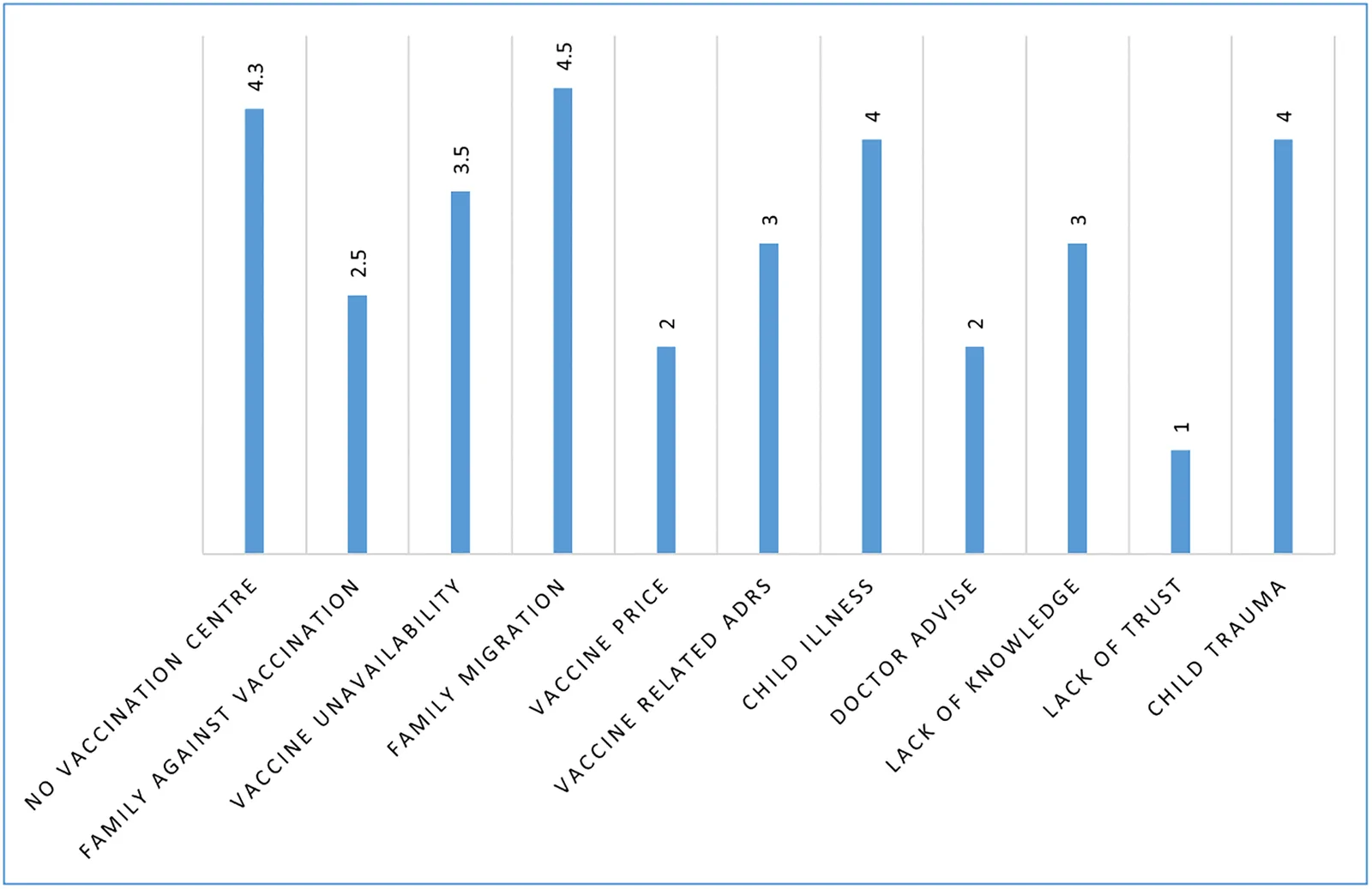
Reasons of immunization delay. Multiple reasons could be reported per caregiver. Lack of knowledge about the vaccination schedule was the most frequently cited reason (54.8%), followed by family opposition to vaccination (19.0%), negative vaccine perception (17.9%), vaccine price (16.1%), and concerns about vaccine-related adverse drug reactions (16.1%). Error bars represent 95% confidence intervals.

### Coverage versus timeliness of vaccination

The overall comparison of vaccine coverage and timeliness across the seven essential visits is presented in Fig 5. Overall coverage (timely + delayed) remained high (>96%) for the first nine months of life. However, timeliness dropped sharply after birth, declining from 98.6% at birth to 73.8% at 2 months, 67.9% at 4 months, 67.3% at 6 months, and reaching its lowest point of 52.4% at 9 months. The largest gap between coverage and timeliness was at the 9-month visit (44.0%), indicating that nearly half of children received the measles vaccine later than recommended. The coverage declined to 85.1% (59.5% timely) at 12-month visit, and at the 18-month visit, coverage dropped further to 63.7% (37.5% timely).

**Fig 5:**
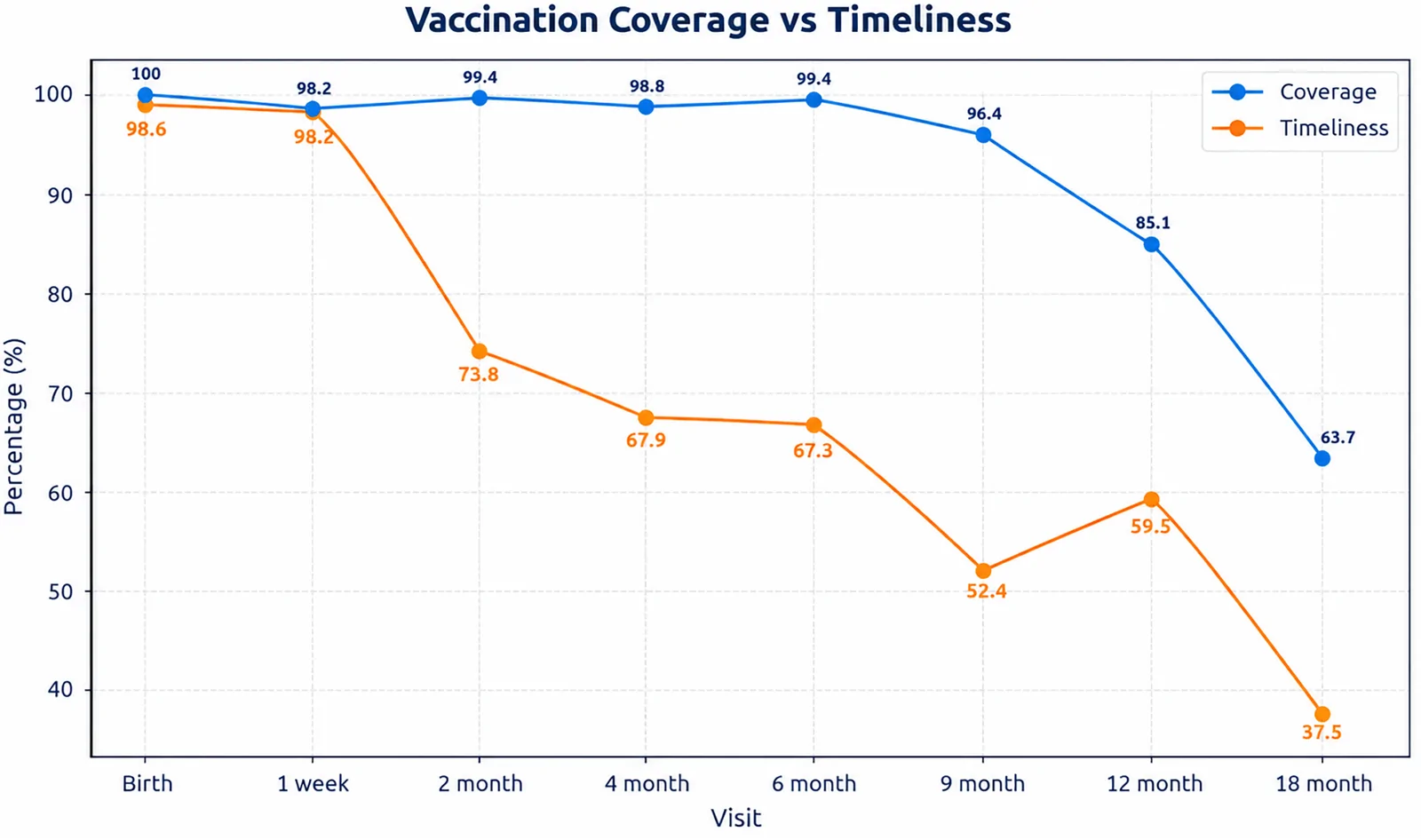
Comparison of overall vaccine coverage and timely vaccination across seven essential immunization visits (n = 168). Coverage (blue circles): proportion of children who received the vaccine (timely or delayed). Timeliness (orange circles): proportion of children who received the vaccine within the recommended 28-day window. The vertical gap between coverage and timeliness represents the proportion of children with delayed vaccination. The largest gap was observed at the 9-month visit (44.0% delayed for measles vaccine), indicating that nearly half of children received this critical vaccine later than recommended. Coverage declined notably after 9 months, dropping to 85.1% at 12 months and 63.7% at 18 months, with timeliness at 59.5% and 37.5%, respectively. Data labels show timely vaccination percentages.

### Factors associated with immunization delay

Bivariate logistic regression analysis was conducted to identify association of factors with delayed immunization (Table 2). In unadjusted analyses, none of the sociodemographic variables demonstrated a statistically significant association with delay in immunization, as all 95% confidence intervals for the odds ratios included the null value of 1.0 (p > 0.05). These variables included maternal age, maternal education, paternal age, paternal education, gender, birth order, place of delivery, or family size.

**Table 2:** Relationship of immunization delay with various factors.

Variables	Categories	Unadjusted odds	Adjusted odds
		(95% CI)	(95% CI)
**Maternal factors**			
Mothers Age	Below 30 years	Ref	
	Between 30–39 years	0.69 (0.25 to 1.89)	
	Between 40–49 years	1.50 (0.15 to 14.35)	
Mothers Education	No education or primary	Ref	
	Secondary	0.54 (0.11 to 2.78)	
	Higher	1.53 (0.27 to 8.52)	
**Paternal factors**			
Fathers Age	Below 30 years	Ref	
	Between 30–39 years	0.76 (0.08 to 6.74)	
	Between 40–49 years	2.17 (0.23 to 20.10)	
Fathers Education	No education or primary	Ref	
	Secondary	0.76 (0.08 to 6.74)	
	Higher	2.17 (0.23 to 20.10)	
**Child factors**			
Child Sex	Male	Ref	
	Female	1.53 (0.62 to 3.78)	
Birth order	Index child	Ref	
	Subsequent child	0.66 (0.26 to 1.64)	
**Household factors**			
Place of birth	Home delivery	Ref	
	Institutional delivery	1.21 (0.14 to 10.23)	
Family size	Small	Ref	
	Large	0.83 (0.28 to 2.42)	
**Refusal reasons**			
Unavailability of vaccine	Yes	Ref	Ref
	No	2.58 (0.72 to 9.18)	4.74 (1.09 to 20.51) *
Migration of the family	Yes	Ref	
	No	3.05 (0.52 to 17.83)	
Child was too sick	Yes	Ref	
	No	1.37 (0.35 to 5.29)	
Did know vaccination schedule	Yes	Ref	Ref
	No	2.55 (0.81 to 8.05)	3.85 (1.04 to 14.24) *
Fear against vaccination	Yes	Ref	
	No	1.12(0.30 to 4.21)	
**Vaccination timeliness**			
1 months	Valid	Ref	
	Delayed	0.19 (0.02 to 1.48)	
2 months	Valid	Ref	
	Delayed	0.57 (0.18 to 1.79)	
4 months	Valid	Ref	
	Delayed	0.92 (0.33 to 2.54)	
6 months	Valid	Ref	
	Delayed	1.44 (0.57 to 3.63)	
9 months	Valid	Ref	
	Delayed	1.41 (0.57 to 3.49)	
12 months	Valid	Ref	
	Delayed	0.34 (0.09 to 1.22)	
18 months	Valid	Ref	
	Delayed	0.70 (0.21 to 2.34)	

Note: CI = Confidence Interval; Ref = Reference category; aOR = Adjusted odds ratio. Multivariate logistic regression model was adjusted for all variables shown in the table. Backward elimination method was used to retain variables with p ≤ 0.05. * Statistically significant at p < 0.05. Adjusted ORs are shown only for variables retained in the final model after backward elimination.

Table 2 presents both unadjusted odds ratios (from bivariate analysis) and adjusted odds ratios from the final multivariate logistic regression model. Adjusted odds ratios are presented only for variables that were retained in the final model after backward elimination. Among the refusal reasons, two factors, vaccination access and caregivers who did not know the vaccination schedule were significant predictors in the adjusted multivariate logistic regression model. After controlling for potential confounding factors, caregivers who reported no issues with vaccine availability had 4.74 times higher odds of having a child with delayed immunization compared to caregivers who cited stockouts as a barrier (a OR = 4.74; 95% CI: 1.09–20.51; p = 0.038). Additionally, caregivers who did not know the vaccination schedule had 3.85 times higher odds of delay compared to those familiar with the schedule (aOR = 3.85; 95% CI: 1.04–14.24; p = 0.043).

There were no significant associations between immunization delay and the timing of any specific vaccination visit (1 month, 2 months, 4 months, 6 months, 9 months, 12 months, or 18 months) in either the unadjusted or adjusted models. Similarly, other refusal reasons that included family migration, child illness, and fear of vaccination were also not significantly associated with immunization delay.

## Discussion

This study explored the determinants of pediatric immunization status among young children in Baghdad, Iraq, focusing on immunization timeliness, missed vaccinations and factors associated with delayed vaccination. To our knowledge, this is the first study in Baghdad, to provide a detailed assessment of immunization timeliness across all essential visits using immunization cards and distinguish between single-visit delays, multiple-visit delays, and missed doses. The findings reveal a high prevalence of immunization delay (91.7%), with the children mostly experiencing delays at multiple visits. Missed vaccinations were highest at 18-month (36.3%). The most common reason for delay was lack of knowledge about the vaccination schedule (54.8%), and the multivariate analysis demonstrated, lack of knowledge (aOR=3.85) and perceived vaccine availability (aOR=4.74) were significantly associated with immunization delay. No sociodemographic factors of the family were significantly associated with delay in the adjusted model.

In this study the finding that only 8.3% of children received all scheduled vaccinations within the recommended timeframe is concerning but it is consistent with previous studies from Iraq and other conflict-affected settings. Molan in 2020 from Basrah, Iraq, reported that 12% of children received vaccines on time [6], similar suboptimal vaccination timeliness has been reported in studies from other post-conflict settings, often due to disruptions in healthcare services and caregiver displacement [12,21]. A notable finding is that 75% of children experienced delays at multiple visits, compared to only 16.7% who were delayed at a single visit. This suggests that when a child falls behind schedule, catching up on the subsequent doses becomes increasingly difficult, highlighting the importance of early intervention and reminder systems to prevent early delays from cascading into cumulative non-compliance [20].

The observed 91.7% delay rate in this study is higher than recent studies from other Iraqi provinces, a study in Hilla, Iraq, reported 61% of children having missed immunization opportunities [22], and a study from Basrah reported good coverage but high defaulter rates [23]. These rates in Baghdad may reflect challenges related to densely populated urban setting which may include, higher caregiver mobility, different priorities, and high expectations for timely vaccination. On the contrary, this discrepancy can be due to different methodology: use of strict 28-day window to define timeliness and documented immunization cards, whereas some studies relied on maternal recall or described coverage rather than timeliness. Suggesting that delay rates in the country may be substantially higher than reported previously if the timeliness is rigorously assessed.

Visit-specific patterns shown a clear decline in adherence with age, early visits (birth till 6 months) had high rates of timely vaccination (67.3% to 98.6%), but timely vaccination dropped with the increasing age. The highest rate of delayed vaccinations was observed at 9-month age (44.0%), and missed vaccination peaked at 18-months (36.3%). This pattern suggests competing priorities, reduced perceived risk of vaccine-preventable diseases by caregivers, or difficulties accessing healthcare facilities that may contribute to declining adherence, observed in other LMICs [24].

Despite high over all coverage (>96%) in the first nine months, timeliness of vaccination was suboptimal. At 2-month, coverage was 99.4%, but only 73.8% received timely vaccination, leaving a quarter of children unprotected. The largest gap was observed at 9 months (44% delay of measles vaccine), a critical period of vulnerability [25]. This finding is alarming given the recent resurgence of measles outbreaks globally and in the Eastern Mediterranean region. Recent data indicate that Iraq has experienced a resurgence of measles outbreaks in 2023–2024 [26]. Coverage dropped sharply after 9 months with only 85.1% coverage at 12 months and 63.7% at 18 months – with missed doses reaching 36.3% at 18 months. The high missed dose rate at 18 months is particularly concerning as this visit includes critical booster doses needed to maintain immunity through early childhood. These findings highlights the need for interventions not only to address early delays but also to target the critical 12–18-month period, where immunization dropout rates are highest [22].

Lack of knowledge related to vaccination schedule was reported by over half of caregivers (54.8%), aligning with previous study in Iraq, where inadequate awareness of parents was identified as a major barrier to childhood immunization [27]. This finding suggest that healthcare providers may not be adequately communicating the timing and importance of follow-up visits. In this study the other reasons of immunization delays (i.e., family opposition to vaccination 19.0%, negative vaccine perception 17.9%, and concerns about vaccine related ADRs 16.1%) highlights the need of health education and family engagement to address the knowledge gaps and barriers due to attitude [1]. Surprisingly vaccine price was identified as a barrier despite vaccines being provided free through the Iraqi EPI, suggesting that indirect costs (transportation and time off work) may still affect timely vaccination [13].

There was a significant association of lack of knowledge of vaccination schedule and perceived vaccine availability with immunization delay. Caregivers not aware of the vaccination schedule had nearly four times higher odds of delayed vaccination (aOR = 3.85; 95% CI: 1.04–14.24), this finding is consistent with studies from other LMICs, where maternal knowledge was a strong predictor of vaccination completion [28–30]. The persistence of this association after controlling education shows that vaccination knowledge is a distinct modifiable factor that can be improved through targeted health education. Caregivers who reported no issues with availability of vaccine had 4.74 times higher odds of delayed vaccination compared to those who didn’t cite availability as barrier (aOR = 4.74; 95% CI: 1.09–20.51). This counter intuitive finding should be interpreted with caution. Rather than giving a causal relationship, this unexpected association may reflect reverse causation, measurement error, residual confounding, or small sample size [31–33]. The wider confidence interval (1.09–20.51) and small number of caregivers who reported availability as barrier (n = 21) may further suggest that the finding may be unstable. This complex relationship between perceived vaccine availability and immunization timeliness needs to be explored further.

Notably, none of the sociodemographic factors showed a significant association with immunization delay, this finding is striking as previous studies reported associations between parental education and vaccination outcomes [24]. The absence of such associations may reflect that people living in urban Baghdad, have almost similar access to information and healthcare services demographic subgroups, or vaccination status is mediated by knowledge and access of vaccination. This finding suggests that strategies focused on caregiver knowledge and vaccine availability may be effective across diverse populations without specific demographic targets.

Based on the key findings, the following recommendations are proposed to address delay of childhood immunization among children in Baghdad, Iraq:

- Implement health education at first visit with printed schedules, reminders, and reinforcing key messages about timely vaccination.
- Ensuring consistent vaccine supply and communicating availability transparently, particularly to 12.5% of caregivers who cited stockouts.
- Community-based interventions engaging parents, and family members to address vaccine hesitancy at the household level.
- Conduct qualitative studies to explore the reasons behind knowledge gaps and the complex relationship between perceived vaccine availability and delay.
- Targeted catch-up vaccination campaigns for children aged 12–24 months, including dedicated vaccination days, home visits, or mobile vaccination units to reach families who have fallen behind schedule.

This study has several notable strengths. Immunization status was assessed using immunization cards to ensure data collection and minimizing recall bias. The enrolment of children aged 18–24 months allowed complete assessment of all seven essential immunization visits [6,34]. Finally, the study was conducted in Baghdad, urban capital of Iraq, providing insights relevant to a major metropolitan setting in a post-conflict context [12]. Despite these strengths, this study possess several limitations. Because of the cross-sectional design, the findings only reflects correlations at one point in time and do not provide causality. The use of purposive sampling, with only 11 of 30 contacted PHCs participating, significantly limits generalizability to the larger population of Baghdad. Therefore, these findings should be considered as indicative of the challenges in urban PHC settings rather than representative of all communities in Baghdad or Iraq. Post-hoc analysis showed the study had adequate power (≥80%) to detect the large effects for knowledge (OR = 3.85) and vaccine availability (OR = 4.74), the study however had limited power to detect smaller effect sizes (OR < 2.5), and non-significant sociodemographic findings should be interpreted with this limitation in mind. The sample size of 168 may limit model stability and the ability to detect smaller but potentially meaningful associations, a larger sample would have allowed for more robust multivariable modelling with additional covariates. Reported reasons of delays by caregiver may be subjected to social desirability bias. Additionally, the study does not assess factor related to healthcare providers (e.g., information given by healthcare providers, operating hours of vaccination centre, provider attitudes) that may influence timeliness of vaccination. This study focused exclusively on children aged 18–24 months, not capturing vaccination delays or missed opportunities occurring after 2 years of age, including booster doses scheduled at 4–6 years (DTaP-IPV, MMR) and later childhood vaccines. The high delay rates observed in the second year of life suggest that vaccination compliance may continue to decline with increasing age, which could be explored in future research to show the full trajectory of childhood immunization compliance in this population. The reasons for immunization delay were assessed only from caregiver perspectives. Inputs from healthcare providers, PHC administration, community health workers, and policymakers, can give a comprehensive outlook of systemic and structural barriers to timely vaccination. Future research should employ mixed-methods approaches to capture multi-stakeholder perspectives.

## Conclusion

This study demonstrates that immunization delays are highly prevalent among children in the sampled Baghdad PHCs, and most children experienced delays at multiple visits with a reasonable proportion of children missing doses of vaccines at later visits. Although these findings provide important insights into vaccination challenges in a major urban post-conflict setting, they should not be generalized to all of Baghdad or Iraq. Lack of knowledge related to vaccination schedule and perception about vaccine availability were the key modifiable factors associated with vaccination delay. Notably, no sociodemographic characteristics were observed as significant predictors of delays, suggesting interventions focusing on knowledge improvement and smooth supply of vaccines may be effective across diverse populations. As Iraq is continuing to rebuild its healthcare system after decades of internal and external conflicts, strengthening immunization services through targeted education, access to vaccination, and community/family engagement will be essential to achieving high vaccination coverage to reduce the burden of vaccine-preventable diseases among children.

## Supporting information

S1 File Study Questionnaire. The pre-validated structured questionnaire used for data collection (adopted and adapted from Khaliq et al., 2017).
(DOCX)

S2 File Anonymized Dataset. Anonymized data in Excel format.
(DOCX)

S3 File STROBE Checklist. Completed STROBE checklist for cross-sectional studies.
(DOCX)

S4 File Data Dictionary. Variable definitions and coding scheme.
(XLSX)
