# Supplementary material for: Immunization timeliness and its determinants among young children in Baghdad, Iraq: A cross-sectional study using documented vaccination cards
Source: PLoS One. 2026 Oct 1;21(10):e0359962. doi: 10.1371/journal.pone.0359962 (PMC13630201; doi:10.1371/journal.pone.0359962)
Supplement: Supplementary file 1 [file pone.0359962.s005.pdf]

PLOS One

Immunization Timeliness and Its Determinants Among Young Children in Baghdad, Iraq: A Cross-Sectional Study Using Documented Vaccination Cards

--Manuscript Draft--

**Manuscript Number:**	PONE-D-26-19652
**Article Type:**	Research Article
**Full Title:**	Immunization Timeliness and Its Determinants Among Young Children in Baghdad, Iraq: A Cross-Sectional Study Using Documented Vaccination Cards
**Short Title:**	Immunization Timeliness in Baghdad, Iraq
**Corresponding Author:**	Syed Shaukat Ali Muttaqi Shah, Ph.D.<br>Al-Farahidi University<br>Baghdad, IRAQ
**Keywords:**	Immunization; vaccination delay; timeliness; childhood vaccination; Iraq; Baghdad
**Abstract:**	Background: Childhood immunization is a critical public health intervention, yet timeliness remains a challenge in post conflict affected settings like Iraq. This study assessed timeliness of immunization, missed doses, and associated factors with delay among children aged 18–24 months in Baghdad, Iraq.<br>Methods: A cross-sectional study was conducted in 11 primary healthcare centers in Baghdad between May and July 2025. Data were collected from 168 children using documented immunization cards. Immunization delay was defined as vaccination administered beyond >28 days of the scheduled date. Multivariate logistic regression was used to identify associated factors.<br>Results: Overall vaccine coverage (timely + delayed) remained high (>96%) for first nine months, at 12 and 18 months, coverage declined to 74.4% and 62.5%, respectively. Only 14 children (8.3%) received all vaccinations on time. Immunization delay was observed in 154 (91.7%) children, with 28 (16.7%) of children with delay at a single visit and 126 (75.0%) at multiple visits. Missed doses were highest at 12 months (37.5%) and 18 months (36.3%). Timely vaccination dropped sharply from 98.6% at birth to 52.4% at 9 months, and 36.3% at 18months. The most common reason for delay was lack of knowledge about the vaccination schedule (54.8%). In adjusted analysis, lack of knowledge of schedule (aOR = 3.85; 95% CI: 1.04–14.24) and perceived vaccine availability (aOR = 4.74; 95% CI: 1.09–20.51) were significantly associated with delay. No sociodemographic factors were significant.<br>Conclusion: Immunization delay is highly prevalent among children in Baghdad. Interventions focusing on caregiver knowledge and ensuring consistent vaccine supply are urgently needed, particularly for children in the second year of life.
**Order of Authors:**	Syed Shaukat Ali Muttaqi Shah, Ph.D.
	Hakim Ahmed Mikhlif
	Mohammed Abdul Wahab Alwardi
	Ale Zehra
	Uroosa Kanwal
	Tehreem Ansari
**Additional Information:**	
**Question**	**Response**
**Financial Disclosure**<br><br>Enter a financial disclosure statement that describes the sources of funding for the work included in this submission. Review the submission guidelines for detailed requirements. View published research	The author(s) received no specific funding for this work.

articles from *PLOS ONE* for specific examples.<br><br>This statement is required for submission and **will appear in the published article** if the submission is accepted. Please make sure it is accurate.<br><br>**Funded studies**<br>Enter a statement with the following details:<br>• Initials of the authors who received each award<br>• Grant numbers awarded to each author<br>• The full name of each funder<br>• URL of each funder website<br>• Did the sponsors or funders play any role in the study design, data collection and analysis, decision to publish, or preparation of the manuscript?<br><br>Did you receive funding for this work?	
**Competing Interests**<br><br>Use the instructions below to enter a competing interest statement for this submission. On behalf of all authors, disclose any competing interests that could be perceived to bias this work—acknowledging all financial support and any other relevant financial or non-financial competing interests.<br><br>This statement is required for submission and **will appear in the published article** if the submission is accepted. Please make sure it is accurate and that any funding sources listed in your Funding Information later in the submission form are also declared in your Financial Disclosure statement.<br><br>View published research articles from *PLOS ONE* for specific examples.	The authors have declared that no competing interests exist.

**NO authors have competing interests**<br><br>Enter: *The authors have declared that no competing interests exist.*<br><br>**Authors with competing interests**<br><br>Enter competing interest details beginning with this statement:<br><br>*I have read the journal's policy and the authors of this manuscript have the following competing interests: [insert competing interests here]*<br><br>* typeset	
**Ethics Statement**<br><br>Enter an ethics statement for this submission. This statement is required if the study involved:<br><br>• Human participants<br>• Human specimens or tissue<br>• Vertebrate animals or cephalopods<br>• Vertebrate embryos or tissues<br>• Field research<br><br>Write "N/A" if the submission does not require an ethics statement.<br><br>General guidance is provided below. Consult the submission guidelines for detailed instructions. **Make sure that all information entered here is included in the Methods section of the manuscript.**	This study involving human participants was reviewed and approved by the Research Ethics Committee, Al-Farahidi University, College of Pharmacy (Approval No.: Farahidi-AD no: 1-652025). All procedures were performed in accordance with the ethical standards of the institutional research committee and with the 1964 Helsinki Declaration and its later amendments. Written informed consent was obtained from the parent or legal guardian of each participating child prior to data collection. Consent forms included information about the study purpose, data confidentiality, and the right to withdraw at any time without consequence. No identifiable personal information was collected, and all data were anonymized prior to analysis. This study did not involve animal subjects.

**Format for specific study types**<br><br>**Human Subject Research (involving human participants and/or tissue)**<br>• Give the name of the institutional review board or ethics committee that approved the study<br>• Include the approval number and/or a statement indicating approval of this research<br>• Indicate the form of consent obtained (written/oral) or the reason that consent was not obtained (e.g. the data were analyzed anonymously)<br><br>**Animal Research (involving vertebrate animals, embryos or tissues)**<br>• Provide the name of the Institutional Animal Care and Use Committee (IACUC) or other relevant ethics board that reviewed the study protocol, and indicate whether they approved this research or granted a formal waiver of ethical approval<br>• Include an approval number if one was obtained<br>• If the study involved *non-human primates*, add *additional details* about animal welfare and steps taken to ameliorate suffering<br>• If anesthesia, euthanasia, or any kind of animal sacrifice is part of the study, include briefly which substances and/or methods were applied<br><br>**Field Research**<br><br>Include the following details if this study involves the collection of plant, animal, or other materials from a natural setting:<br>• Field permit number<br>• Name of the institution or relevant body that granted permission	
Data Availability<br><br>Before publication, Authors are required to make fully available and without restriction all data underlying their findings. Sharing the data underlying your findings increases the visibility, credibility, and impact of your work, while supporting transparency and future scientific discovery. Please see our PLOS Data Policy page for detailed information on this policy.</p> A Data Availability	The anonymized dataset supporting the findings of this study is available within the paper and its Supporting Information files (S2 File). The data have been anonymized to protect participant confidentiality. The original data are available from the corresponding author upon reasonable request.

Statement, detailing where the data can be accessed, is required at first submission. Insert your Data Availability Statement in the box below.</p><br><br>Please see the data reporting of our submission guidelines for instructions on what you need to include in your Data Availability Statement. Examples of acceptable Data Availability Statements are included here for reference:</p><br> <br> - The minimal data set [and accompanying code, where generated] is available at [repository name] via [DOI hyperlink] <br> - No data was generated by this study. The following existing data sources were used: [dataset name] from [dataset location] available via [web address or DOI hyperlink]. <br> - For Study Protocols: No datasets were generated or analysed during the current study. All relevant data from this study will be made available upon study completion. <br> <br><br><i>PLOS allows rare exemptions to address legal and ethical concerns. If you have legal or ethical restrictions, please use the box below to detail these in full sentences for the Journal team to consider.</i></p><br><br>This statement will be typeset if the manuscript is accepted for publication.</p>	

Powered by Editorial Manager® and ProduXion Manager® from Aries Systems Corporation

Manuscript
Click here to access/download;Manuscript;Manuscript vaccination 16042026.docx

Immunization Timeliness and Its Determinants Among Young Children in Baghdad, Iraq: A Cross-Sectional Study Using Documented Vaccination Cards

Authors:

Syed Shaukat Ali Muttaqi Shah[1*], Hakim Ahmed Mukhlif[2], Mohamed Abdul Wahab Alwardi[1], Ale Zehra[3], Uroosa Kanwal[4], Tehreem Ansari[5]

Affiliations:

1. Department of Clinical Pharmacy, College of Pharmacy, Al-Farahidi University, Baghdad, Iraq
2. Department of Pharmacognosy, College of Pharmacy, Al-Farahidi University, Baghdad, Iraq
3. Department of Pharmacy Practice, Faculty of Pharmaceutical Sciences, Dow College of Pharmacy, Dow University of Health Sciences, Karachi, Sindh, Pakistan
4. Faculty of Pharmacy, Salim Habib University, Karachi, Pakistan
5. Department of Infectious Disease, Dow University Hospital (Ojha Campus), Dow University of Health Sciences, Karachi, Sindh, Pakistan

*Corresponding author: Syed Shaukat Ali Muttaqi Shah: syedshaukatali@uoalfarahidi.edu.iq

Abstract

Background: Childhood immunization is a critical public health intervention, yet timeliness remains a challenge in post conflict affected settings like Iraq. This study assessed timeliness of immunization, missed doses, and associated factors with delay among children aged 18–24 months in Baghdad, Iraq.

Methods: A cross-sectional study was conducted in 11 primary healthcare centers in Baghdad between May and July 2025. Data were collected from 168 children using documented immunization cards. Immunization delay was defined as vaccination administered beyond >28 days of the scheduled date. Multivariate logistic regression was used to identify associated factors.

Results: Overall vaccine coverage (timely + delayed) remained high (>96%) for first nine months, at 12 and 18 months, coverage declined to 74.4% and 62.5%, respectively. Only 14 children (8.3%) received all vaccinations on time. Immunization delay was observed in 154 (91.7%) children, with 28 (16.7%) of children with delay at a single visit and 126 (75.0%) at multiple visits. Missed doses were highest at 12 months (37.5%) and 18 months (36.3%). Timely vaccination dropped sharply from 98.6% at birth to 52.4% at 9 months, and 36.3% at 18months. The most common reason for delay was lack of knowledge about the vaccination schedule (54.8%). In adjusted analysis, lack of knowledge of schedule (aOR = 3.85; 95% CI: 1.04–14.24) and perceived vaccine availability (aOR = 4.74; 95% CI: 1.09–20.51) were significantly associated with delay. No sociodemographic factors were significant.

Conclusion: Immunization delay is highly prevalent among children in Baghdad. Interventions focusing on caregiver knowledge and ensuring consistent vaccine supply are urgently needed, particularly for children in the second year of life.

Keywords: Immunization, vaccination delay, timeliness, childhood vaccination, Iraq, Baghdad

Introduction

Vaccination is one of the most important public health interventions in the history of medicine, providing primary prevention of serious infections [1]. Globally, vaccinations are estimated to prevent around 2 to 3 million deaths every year [2]. Childhood vaccination is the cornerstone of public health interventions, playing a vital role in preventing infectious diseases, and significantly reducing morbidity and mortality rates [1, 3]. The World Health Organization (WHO) established the expanded program for immunization (EPI) has significantly reduced vaccine-preventable diseases and deaths since 1974 [4]. EPI aims to extend new vaccines and preventive health interventions to all the parts of the world, originally the program was focused on six diseases (tuberculosis, diphtheria, pertussis, polio and measles), it has expanded to include 13 universally recommended vaccines, including Covid-19 [5]. These vaccination programs have contributed vitally to the eradication of diseases such as polio, small-pox, measles and tetanus globally [6].

Compliance to childhood vaccination is challenging; globally immunization programs reach over 80% of children under one year of age, preventing around 2.5 million deaths per year, while it is estimated if the coverage increases to 90% it can prevent 2 million more deaths annually [7]. In order to increase the coverage and compliance, a number of actions like scientific advancement in production, simplified delivery methods, uninterrupted supplies of vaccination, and social/political commitment to the vaccination program is highly warranted [8]. On-time vaccination is important to protect children from vaccine preventable diseases, and children who receive vaccination later than recommended schedule remain susceptible to these diseases [9]. Delay in immunization has serious consequences on health systems especially during an outbreak [10].

Iraq had one of the most advanced healthcare systems in the Middle East, achieving universal health from 1960s to 1980s [11]. However, healthcare system has been significantly impacted by the decades of conflict and instability [12]. Despite improvement in the immunization system over the past decade, the country is still struggling to provide the vaccine to all the children making vaccine-preventable diseases as a primary cause of child morbidity and mortality [7]. According to a recent report from the Iraqi Ministry of Health, the Polio, BCG and Measles vaccination rates are above 90%; however 1.5 million children still need to be fully vaccinated, indicating a significant gap between the immunization requirements and actual compliance [13]. Amongst common factors for the low compliance regarding childhood immunization in Iraq, inadequate parental awareness has been identified as one of the major reason [14]. Misconceptions related to safety and efficacy of vaccines, religious beliefs, and lack of trust in government policies also results in noticeable vaccine hesitancy and refusal [6, 15].

This study was conducted in Baghdad, Iraq to assess compliance with the childhood vaccination focusing on children aged 18 to 24 months, as this age group should have completed all seven essential immunization visits recommended by the Iraqi Ministry of Health. The specific aims of the study were to: (1) determine the proportion of children with timely, delayed (single or multiple visits), and missed vaccinations across all seven essential immunization visits (1st week, 2, 4, 6, 9, 12, and 18 months); (2) examine the association of delayed immunization with caregiver knowledge, beliefs, education, socioeconomic status, and family size; and (3) identify reasons for immunization delay.

Q1:
Have the booster doses been taken into consideration?

This study is novel in several aspects. First, it assesses immunization timeliness across all seven essential visits using documented immunization cards rather than maternal recall. Second, it is the first study in Iraq to provide a characterization of non-compliance patterns, distinguished between single or multiple visit delays and missed doses of vaccination. Third, it evaluates the impact of both vaccine availability and caregiver knowledge on immunization delay for children under two years of age in a post-conflict urban setting.

Q2:
An important chunk of delay also happens after 2 years. How has that been addressed?

Q3:
Describe a post conflict Urban setting wrt Health care worker availability, duration since conflict, damage done etc

Methodology:

Study design, setting, and duration:

An observational cross-sectional study was conducted in 11 primary healthcare centres (PHCs) of Baghdad, Iraq, between May 8th 2025 and July 31st 2025. Baghdad is the administrative capital of Iraq and is the most densely populated urban community in Iraq; conducting this study in the city makes it a critical site for evaluating national health initiatives like vaccination programs and can provide insight into challenges within a major metropolitan setting.

Q4: Brief the Health care system in Urban Baghdad.
Do PHCs also exist in urban area? If yes, what is the population it caters to?

Study population and their selection criteria:

The primary purpose of this study was to examine the immunization timeliness among the young children of Baghdad city, Iraq. This study chiefly targeted young children aged between 18 and 24 months, because by the age of 18 months, a child should have received basic immunization proposed by the Iraqi Ministry of Health [16], by the age of 18 months, a child should have made seven essential immunization visits [17]. These seven immunization visits should be carried out at childbirth, second month, fourth month, sixth month, ninth month, 15 months and 18 months of their life [16, 17]. One of the basic criteria of this study participation was to provide documented evidence regarding the child's age and child immunization. Children who failed to present immunization card at the time of data collection were excluded from the study participation irrespective of their physical and biological age. Furthermore, any child who had not reach the age of 18 month at the time of data collection was excluded from the study, because their inclusion may either overestimate or underestimate immunization compliance at any immunization visit. Therefore, this study only considered those children who were eligible to reach the age of seven essential immunization visits, i.e., children aged over 18 months.

Q5: What are the visits at 4 and 6 months for. Brief about the Immunisation schedule under the Iraq Ministry

Sample size:

Sample size was calculated by taking immunization coverage into consideration from previous study in Iraq, that reported 83.7% of measles vaccination [18]. Using OpenEPI software with a 95% confidence interval, a 5.6% margin of error, the minimum required sample was calculated as 168. The calculated sample size provides 80% power to detect a 20% difference in immunization delay at 5% significance level. Furthermore, the sample size exceeded the minimum requirement of 10 event per predictor variable for logistic regression analysis. Therefore, a target sample of 168 participants was considered adequate.

Q6: Calculating Sample size by considering only the measles coverage might lead to miscalculations.
Either take complete immunisation coverage or prevalence of delay.

Sampling method:

While random sampling was not feasible due to logistic constraints and need of prior administrative approval of primary healthcare centres (PHCs), a multistage stratified purposive sampling technique was used. In the first stage, PHCs were purposively selected based on their willingness to grant permission for data collection after formal application for data collection, resulting in 11 participating PHCs out of 30 contacted. In the second stage, participants within each selected PHC were also purposively approached based on the convenience and availability at the time of data collection.

Q7: Describe properly the multistage startification that was applied?

Data collection tool:

In this study, the data was collected using a pre-validated and structured questionnaire, which was adopted from Khaliq et al. (2017) [19] and is provided as "S1 File". The questionnaire used in this study consist of 63 questions, which were spread into seven different sections. Each section has different components. The first three sections of the questionnaire were related to the participant's screening, eligibility, consent and enrolment, while the last four sections asked questions related to participant demography, participant knowledge and behaviour, participant immunization status at each immunization visit, collected vaccination dates from cards, and perceived reason of immunization delay.

Study variables:

The immunization status of children aged at 18 months or over was assessed by inspecting their immunization card. Following immunization card assessments, we derived two different types of immunizations outcomes: valid immunization and delayed immunization.

Children who received age-proportionate vaccination on time or within the 28 days window were classified to have *valid immunization*, while any children who received immunization beyond 28 days window periods were categorized to have *delayed immunization*. The delayed immunization was of two types – *immunization delayed at a single visit and immunization delayed at multiple visits*. Apart from immunization timing, various maternal, paternal, household and child factors were also taken into consideration.

The maternal factors include maternal age (less than 20 years, 20 to 29 years, 30 to 39 years and 40 to 49 years) and maternal education (no education, primary, secondary, and higher). Similarly, the *paternal factors:* The paternal factors include paternal age (less than 20 years, 20 to 29 years, 30 to 39 years and 40 to 49 years) and paternal education (no education, primary, secondary, and higher). However, child sex (male and female) and birth order (first birth and subsequent birth) were considered as child factors. The household factors comprise of family size (small family and medium to large family) and type of place of delivery (home delivery and institutional delivery). Households having family size of five or less than five members were classified as small family sized households, whereas those with over five family members were grouped into medium to large sized households. Similarly, the place of delivery was also bifurcated into two major groups: institutional delivery and home delivery. Children born in any public and private clinic or hospital or maternity centre or basic health units were grouped as institutional delivery, while those born in their own or their relative or neighbour or friends house were classified to have home delivery. The data dictionary defining all variables and coding is available as "S2 File".

Data collection procedure and management:

The data of this study was collected by a team of pharmacy final year students studying at Al-Farahidi University. All the students received a formal training of two weeks from their supervisor. The training included how to approach potential participants, describing the study purpose, pre-enrolment screening, eligibility determination, consent taking procedure, and enrolment. Moreover, all the data collectors also received soft skills training for data collection, such as how to approach, how to greet, and how to conclude the interview. The project supervisor divided different tasks to a groups of data collector each week, which include data collection, data entry, and data quality assurance (spot-checks and double data entry) for reducing the unintentional missing data and for improving the quality and transparency of the collected data.

Q8: How was missing data addressed?

Data analysis:

The data of this study was analysed at three different steps. At first, preliminary analysis was performed to examine the distribution of each categorical and numerical variables. Secondly, descriptive statistics was performed in which we calculated the percentages of all the categorical variables, and finally inferential statistics was performed using bivariate logistic regression. Children who received immunization on time were considered as reference, while those who had delayed immunization were tested against the reference. A confidence interval of 95% and a p-value of ≤ 0.05 was considered as significant value for assessing the association of delay immunization with different covariates. Furthermore, multivariate logistic regression was performed but only variable with p-value ≤ 0.2 or less were considered in the model. A backward elimination method was adopted, in which variables with p-value > 0.05 were removed sequentially until the final model contains only a few variables with p-value ≤ 0.05. For demographic and refusal reason variables, missing responses were recoded as 0 (indicating absence of the barrier). Vaccination status variables, only children with complete immunization card

data were included in the analysis, as incomplete cards were an exclusion criterion. No imputation methods were applied due to the low proportion of missing data (<5% for all variables)

Ethical considerations:

This study was approved by the Research Ethics Committee, Al-Farahidi University, college of Pharmacy, approval number Farahidi-AD no: 1-652025. Furthermore, a formal permission from each primary healthcare centre was also taken prior to the study data collection. A written consent from parents/guardian of the child was obtained, and a complete information about the study purpose, and participant's rights was provided, including data confidentiality, anonymity, and rights to refuse or withdraw from participation. In addition, risk and benefits associated with the study participation was also provided to each participant before their participation and enrolment. The STROBE checklist for cross-sectional studies is provided as "S3 File".

The overall study methodology and participant selection process are illustrated in Figure 1

Figure 1: Study flowchart of participant selection and methodology

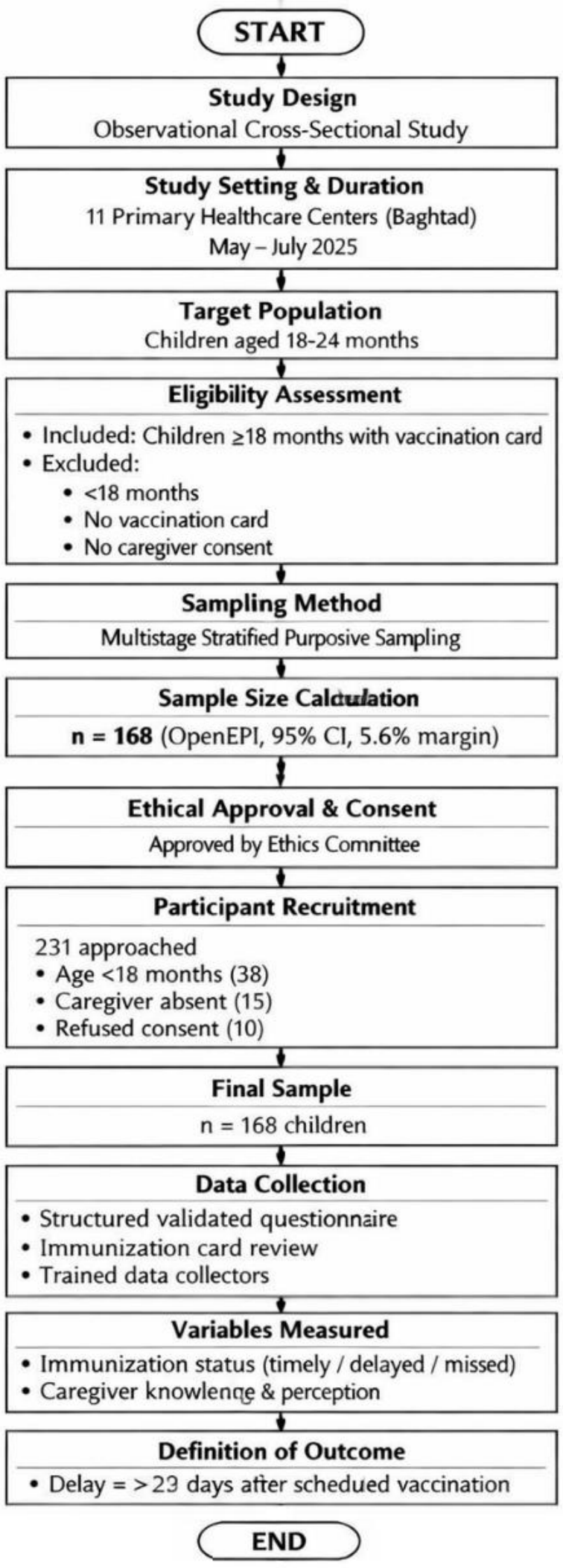

Results:

Descriptive Analysis of Study Participants:

A total of 231 participants were approached, of these, 63 were excluded from the final analysis. The reasons included: 38 children were below 18 months of age; 15 children excluded because primary caregiver was not present at the time of data collection; and 10 caregivers did not provide consent for study participation, resulting in a final sample size of 168 participants included in this study.

Among these 168 participants, more than half of mothers were aged 20–29 years (61.3%) followed by 30–39 years (34.5%), while less than 5% mothers were of aged between 40–49 years and below 20 years, with the mean (±SD) age 28.4 ± 5.7 years. Regarding maternal education, 6.5% had no formal education, 23.2% had primary education, 50% had secondary education, and 20.2% had higher education. Among fathers, 56% were aged 30–39 years, 33.9% were 20–29 years, and 10.1% were 40–49 years. None were reported to be below 20 years. In terms of paternal education, 4.2% had no education, 25% had primary education, 48.2% had secondary education, and 22.6% had higher education.

Of the children, 54.8% were male and 45.2% were female. Regarding birth order, 36.9% were index children and 63.1% were subsequent children. Most children were born in health institutions (94.6%), while 5.4% were delivered at home. In terms of family size, 48.2% of households were categorized as small, and 51.8% as medium or large.

Table-1: Demographic characteristics of the study sample. Q9: May remove or merge the rows with too less values [< 5]

Variables	Categories	N	Percentage
Mothers Age	Below 20 years	2	1.2%
	Between 20 to 29 years	103	61.3%
	Between 30 to 39 years	58	34.5%
	Between 40 to 49 years	5	3%
Mothers Education	No education	11	6.5%
	Primary	39	23.2%
	Secondary	84	50%
	Higher	34	20.2%
Fathers Age	Below 20 years	0	-
	Between 20 to 29 years	57	33.9%
	Between 30 to 39 years	93	55.4%
	Between 40 to 49 years	17	10.1%
Fathers Education	No education	7	4.2%
	Primary	42	25%
	Secondary	81	48.2%
	Higher	38	22.6%
Gender	Male	92	54.8%
	Female	76	45.2%
Birth order	Index child	62	36.9%
	Subsequent child	106	63.1%
Place of birth	Home delivery	9	5.4%
	Institutional delivery	159	94.6%
Family size	Small	81	48.2%
	Medium OR large	87	51.8%

Note: Data are presented as frequencies (N) and column percentages. Percentages may not sum to 100% due to rounding. CI = Confidence Interval. SD = Standard Deviation

Immunization Timeliness and Coverage:

Assessment of vaccination cards revealed that 154 children (91.7%; 95% CI: 86.5-95.2) experienced delayed vaccination at least at one or multiple occasions. Out of these, 28 children (16.7%) were delayed at a single visit, while 126 children (75.0%) experienced delays at multiple visits. The remaining 14

children (8.3%) received all scheduled vaccinations within the recommended timeframes or permissible 28 days window, elaborated in **Figure 2.**

Figure 2: Immunization timeliness (n=168)

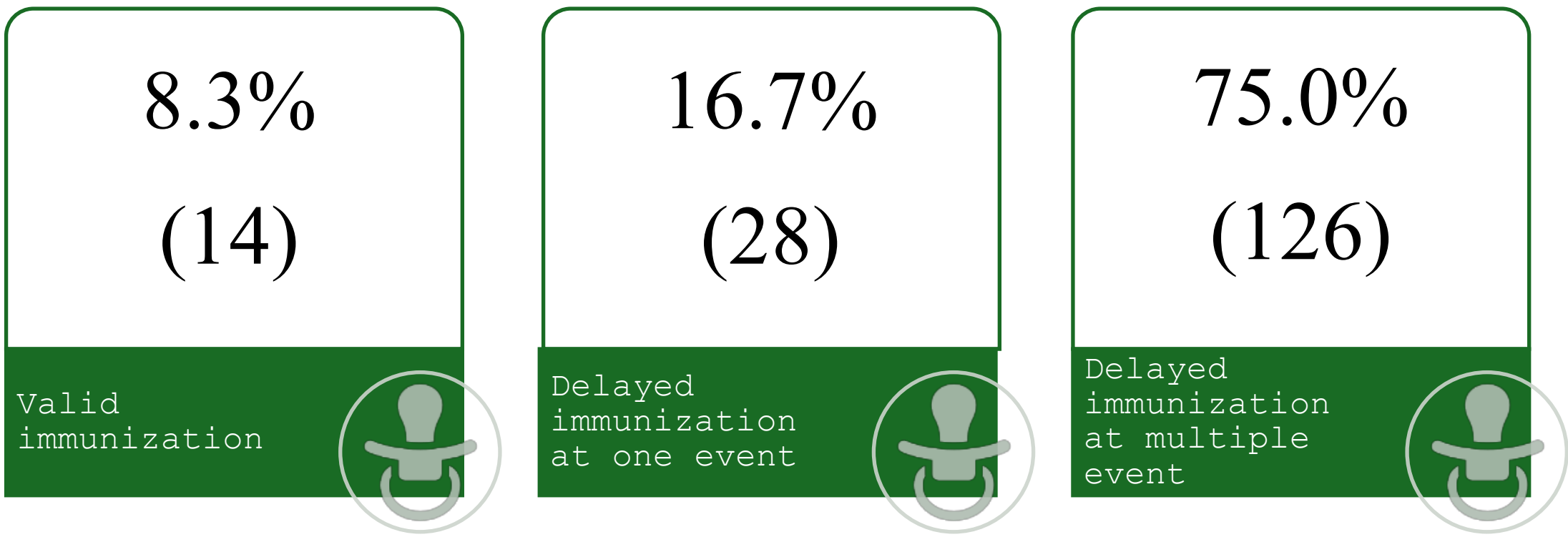

Figure 2 captions: Only 14 children (8.3%) received all scheduled vaccinations within the recommended 28-day window. A total of 154 children (91.7%) experienced delays, with 28 (16.7%) delayed at a single visit and 126 (75.0%) delayed at multiple visits Q10: Is the multiple visit delay inclusive of the single delays?

Age-Proportionate Immunization Status: Q11: What was the cut off for missed vaccination & differentiate it well from the Delays?

The proportion of children with timely, delayed, and missed vaccinations across seven visits is elaborated in **Figure 3**. At the first-week visit, 138 children (82.1%; 95% CI: 75.6–87.5) received timely vaccination, 30 children (17.9%; 95% CI: 12.6–24.4) experienced delays. At the 2-month visit, 124 children (73.8%; 95% CI: 66.6–80.1) received timely vaccination, 43 children (25.6%; 95% CI: 19.4–32.7) experienced delays. At 4-month visit, 114 children (67.9%; 95% CI: 60.5–74.6) received vaccination timely, 52 children (31.0%; 95% CI: 24.2–38.4) received delayed. At the 6-month visit, 113 children (67.3%; 95% CI: 59.9–74.1) were timely, 54 children (32.1%; 95% CI: 25.3–39.6) delayed. At the 9-month visit, 88 children (52.4%; 95% CI: 44.8–59.9) received timely vaccination, 74 children (44.0%; 95% CI: 36.6–51.7) were delayed, and 13 children (7.8%; 95% CI: 4.4–13.0) missed the dose. At the 12-month visit, 100 children (59.5%; 95% CI: 51.9–66.8) were timely, 25 children (14.9%; 95% CI: 10.1–21.0) delayed, and 63 children (37.5%; 95% CI: 30.4–45.0) missed. At the 18-month visit, 61 children (36.3%; 95% CI: 29.2–43.9) received timely vaccination, 44 children (26.2%; 95% CI: 19.9–33.4) were delayed, and 6 children (3.6%; 95% CI: 1.5–7.6) missed the dose.

Figure 3: Age proportionate immunization status of children below two years.

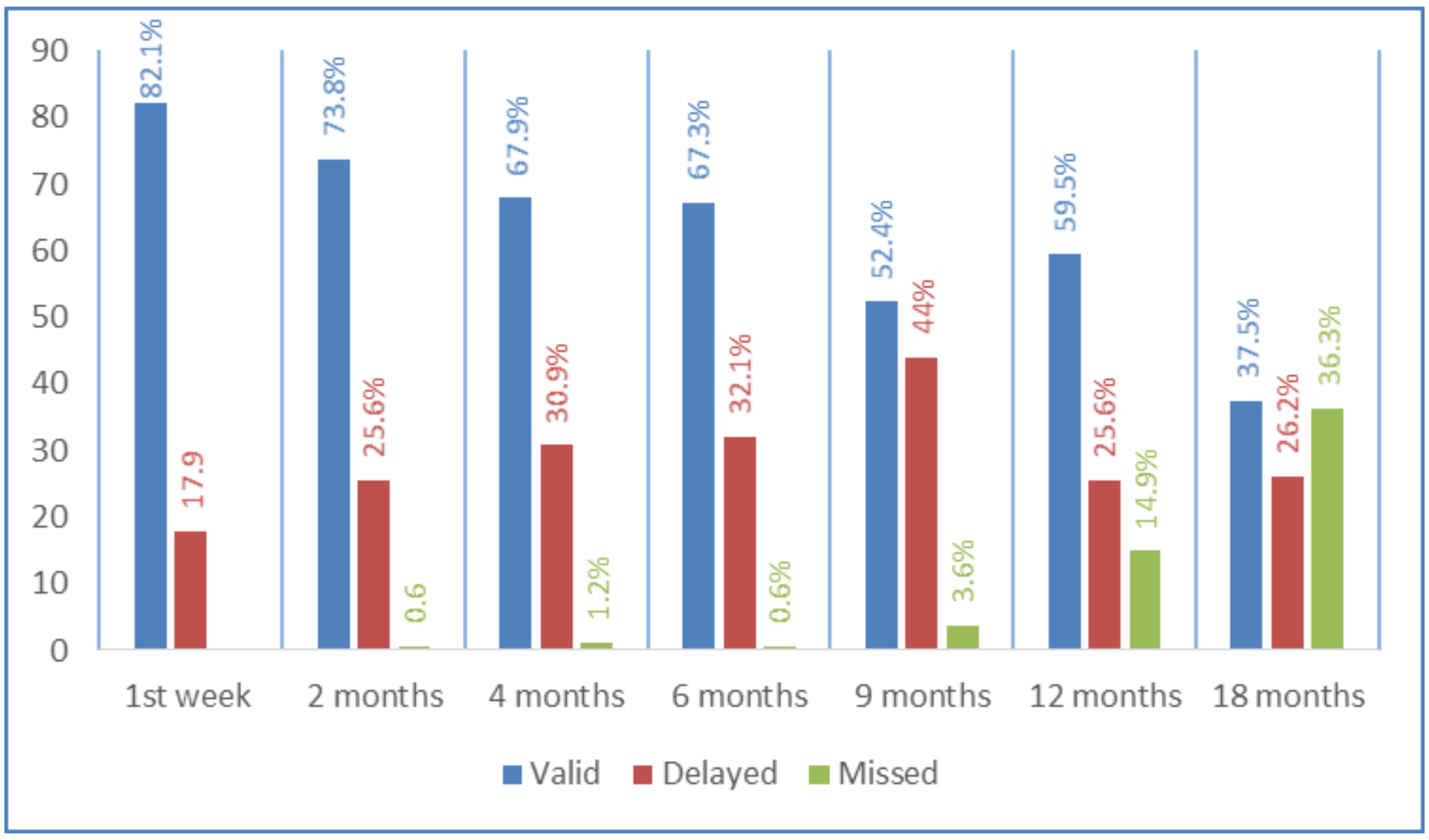

Figure 3 captions: The figure shows the proportion of children with timely, delayed, and missed vaccinations at each scheduled visit (birth, 2, 4, 6, 9, 12, and 18 months). Error bars represent 95% confidence intervals. Timely vaccination declined sharply after birth, from 98.6% at birth to 52.4% at 9 months and 36.3% at 18 months. Missed doses were highest at 12 months (37.5%) and 18 months (36.3%).

Reasons for Immunization Delay:

Reasons for delayed immunization as per the caregivers are presented in **Figure 4**. The most frequently mentioned reason of delayed vaccination was lack of knowledge regarding the vaccination schedule 92 (54.8%; 95% CI: 47.1–62.3). Other reasons included family against vaccination 32 (19.0%; 95% CI: 13.6–25.8), negative perception about vaccination 30 (17.9%; 95% CI: 12.6–24.5), vaccine price 27 (16.1%; 95% CI: 11.1–22.4), and vaccine-related adverse drug reactions (ADRs) 27 (16.1%; 95% CI: 11.1–22.4). Child trauma was reported by 22 caregivers (13.1%; 95% CI: 8.6–19.1), vaccine availability issues by 21 (12.5%; 95% CI: 8.1–18.4), child illness by 16 (9.5%; 95% CI: 5.7–15.0), and family migration was the least frequently cited reason, reported by only 6 caregivers (3.6%; 95% CI: 1.5–7.7).

Figure 4: Reasons of immunization delay

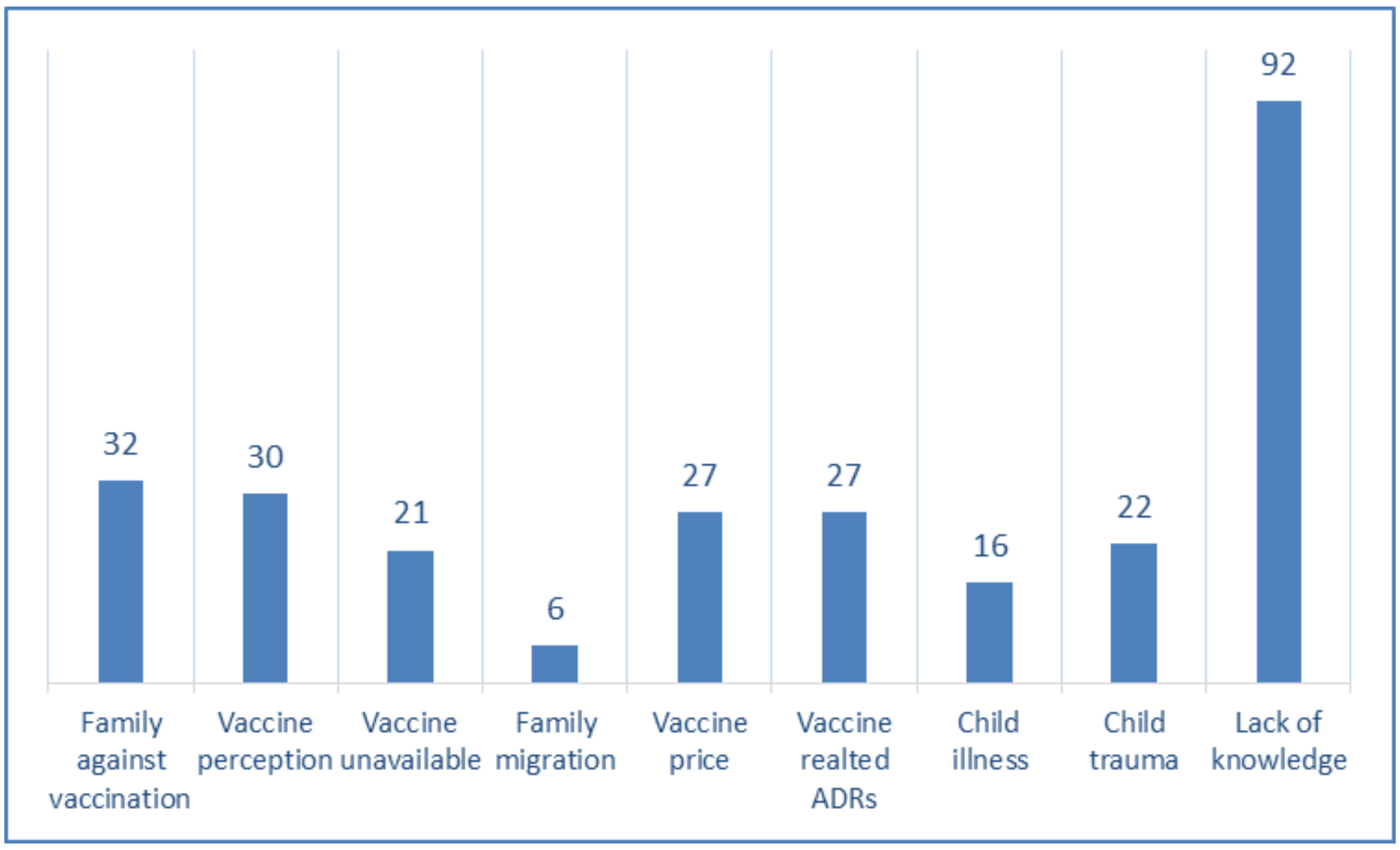

Figure 4 captions: Multiple reasons could be reported per caregiver. Lack of knowledge about the vaccination schedule was the most frequently cited reason (54.8%), followed by family opposition to vaccination (19.0%), negative vaccine perception (17.9%), vaccine price (16.1%), and concerns about vaccine-related adverse drug reactions (16.1%). Error bars represent 95% confidence intervals.

Coverage Versus Timeliness of Vaccination:

The overall comparison of vaccine coverage and timeliness across the seven essential visits is presented in Figure 5. Overall coverage (timely + delayed) remained high (>96%) for the first nine months of life. However, timeliness dropped sharply after birth, declining from 98.6% at birth to 73.8% at 2 months, 67.9% at 4 months, 67.3% at 6 months, and reaching its lowest point of 52.4% at 9 months. The largest gap between coverage and timeliness was at the 9-month visit (44.0%), indicating that nearly half of children received the measles vaccine later than recommended. The coverage declined to 74.4% (59.5% timely) at 12-month visit, and at the 18-month visit, coverage dropped further to 62.5% (36.3% timely).

Figure 5: Comparison of overall vaccine coverage and timely vaccination across seven essential immunization visits (n=168)

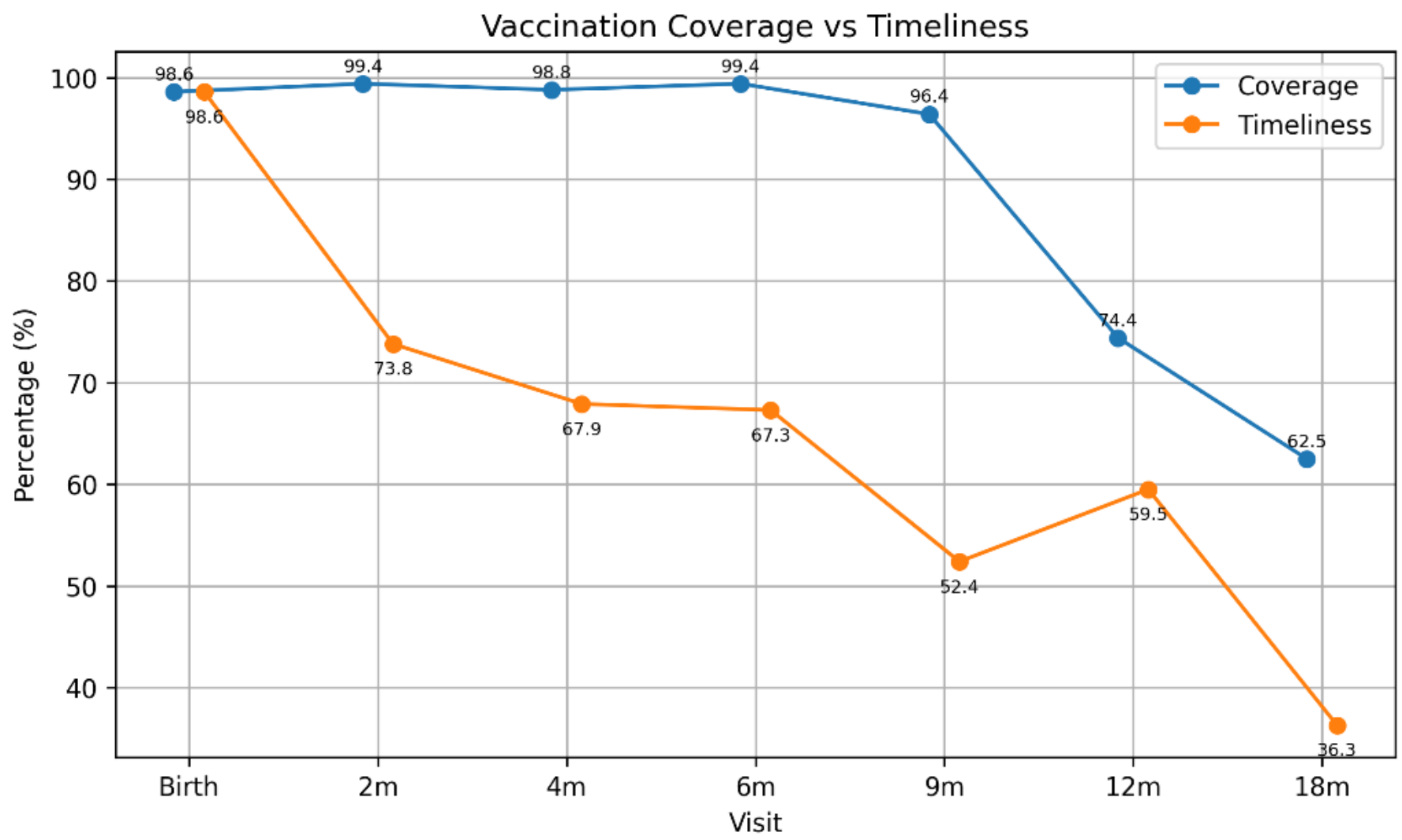

Figure 5 caption: Coverage (blue circles): received vaccine (timely or delayed). Timeliness (orange circles): received within 28-day window. Vertical gap = delay proportion. Largest gap at 9 months (44.0%). Data labels show timely percentages.

Factors Associated with Immunization Delay:

Bivariate logistic regression analysis was conducted to identify association of factors with delayed immunization (Table 2). In unadjusted analyses, none of the sociodemographic variables demonstrated a statistically significant association with delay in immunization, as all 95% confidence intervals for the odds ratios included the null value of 1.0 ($p > 0.05$). These variables included maternal age, maternal education, paternal age, paternal education, gender, birth order, place of delivery, or family size.

Among the refusal reasons, two factors, vaccination access and caregivers who did not know the vaccination schedule were significant predictors in the adjusted multivariate logistic regression model. After controlling for potential confounding factors, caregivers who reported no issues with vaccine availability had 4.74 times higher odds of having a child with delayed immunization compared to caregivers who cited stockouts as a barrier (a OR = 4.74; 95% CI: 1.09–20.51; $p = 0.038$). Additionally, caregivers who did not know the vaccination schedule had 3.85 times higher odds of delay compared to those familiar with the schedule (aOR = 3.85; 95% CI: 1.04–14.24; $p = 0.043$).

There were no significant associations between immunization delay and the timing of any specific vaccination visit (1 month, 2 months, 4 months, 6 months, 9 months, 12 months, or 18 months) in either the unadjusted or adjusted models. Similarly, other refusal reasons that included family migration, child illness, and fear of vaccination were also not significantly associated with immunization delay.

Table-2: Relationship of immunization delay with various factors.

Variables	Categories	Unadjusted odds (95% CI)	Adjusted odds (95% CI)
**Maternal factors**			
Mothers Age	Below 30 years	Ref	
	Between 30 to 39 years	0.69 (0.25 to 1.89)	
	Between 40 to 49 years	1.50 (0.15 to 14.35)	
Mothers Education	No education or primary	Ref	
	Secondary	0.54 (0.11 to 2.78)	
	Higher	1.53 (0.27 to 8.52)	
**Paternal factors**			
Fathers Age	Below 30 years	Ref	
	Between 30 to 39 years	0.76 (0.08 to 6.74)	
	Between 40 to 49 years	2.17 (0.23 to 20.10)	
Fathers Education	No education or primary	Ref	
	Secondary	0.76 (0.08 to 6.74)	
	Higher	2.17 (0.23 to 20.10)	
**Child factors**			
Child Sex	Male	Ref	
	Female	1.53 (0.62 to 3.78)	
Birth order	Index child	Ref	
	Subsequent child	0.66 (0.26 to 1.64)	
**Household factors**			
Place of birth	Home delivery	Ref	
	Institutional delivery	1.21 (0.14 to 10.23)	
Family size	Small	Ref	
	Large	0.83 (0.28 to 2.42)	
**Refusal reasons**			
Unavailability of vaccine	Yes	Ref	Ref
	No	2.58 (0.72 to 9.18)	4.74 (1.09 to 20.51) *
Migration of the family	Yes	Ref	
	No	3.05 (0.52 to 17.83)	
Child was too sick	Yes	Ref	
	No	1.37 (0.35 to 5.29)	
Did know vaccination schedule	Yes	Ref	Ref
	No	2.55 (0.81 to 8.05)	3.85 (1.04 to 14.24) *
Fear against vaccination	Yes	Ref	
	No	1.12(0.30 to 4.21)	
**Vaccination timeliness**			
1 months	Valid	Ref	
	Delayed	0.19 (0.02 to 1.48)	
2 months	Valid	Ref	
	Delayed	0.57 (0.18 to 1.79)	
4 months	Valid	Ref	
	Delayed	0.92 (0.33 to 2.54)	
6 months	Valid	Ref	
	Delayed	1.44 (0.57 to 3.63)	
9 months	Valid	Ref	

	Delayed	1.41 (0.57 to 3.49)	
12 months	Valid<br>Delayed	Ref<br>0.34 (0.09 to 1.22)	
18 months	Valid<br>Delayed	Ref<br>0.70 (0.21 to 2.34)	
Note: CI = Confidence Interval; Ref = Reference category; aOR = Adjusted odds ratio. Multivariate logistic regression model was adjusted for all variables shown in the table. Backward elimination method was used to retain variables with $p \leq 0.05$. $p < 0.05$ indicates statistical significance			

Q12: Why havent the adjusted Odds ratio for rest of the factors taken out...Need a mention on the factors that were taken forward and the cut off used.

Discussion:

This study explored the determinants of pediatric immunization status among young children in Baghdad, Iraq, focusing on immunization timeliness, missed vaccinations and factors associated with delayed vaccination. To our knowledge, this is the first study in Baghdad, to provide a detailed assessment of immunization timeliness across all essential visits using immunization cards and distinguish between single-visit delays, multiple-visit delays, and missed doses. The findings reveal a high prevalence of immunization delay (91.7%), with the children mostly experiencing delays at multiple visits. Missed vaccinations were particularly high at the 12-month (37.5%) and 18-month (36.3%) visits. The most common reason for delay was lack of knowledge about the vaccination schedule, and the multivariate analysis demonstrated, lack of knowledge and vaccine availability were significantly associated with immunization delay. No sociodemographic factors of the family were significantly associated with delay in the adjusted model.

Q13: The perspective of multiple stakeholders in finding out the reasons for delay would provide better cues

In this study the finding that only 8.3% of children received all scheduled vaccinations within the recommended timeframe is concerning but it is consistent with previous studies from Iraq and other conflict-affected settings. Molan in 2020 from Basrah, Iraq, reported that 12% of children received vaccines on time [6], similar suboptimal vaccination timeliness has been reported in studies from other post-conflict settings, often due to disruptions in healthcare services and caregiver displacement [12, 20]. A notable finding is that 75% of children experienced delays at multiple visits, compared to only 16.7% who were delayed at a single visit. This suggests that when a child falls behind schedule, catching up on the subsequent doses becomes increasingly difficult, highlighting the importance of early intervention and reminder systems to prevent early delays from cascading into cumulative non-compliance [21].

The observed 91.7% delay rate in this study is higher than recent studies from other Iraqi provinces, a study in Hilla, Iraq, reported 61% of children having missed immunization opportunities [22], and a study from Basrah reported good coverage but high defaulter rates [23]. These rates in Baghdad may reflect challenges related to densely populated urban setting which may include, higher caregiver mobility, different priorities, and high expectations for timely vaccination. On the contrary, this discrepancy can be due to different methodology: use of strict 28-day window to define timeliness and documented immunization cards, whereas some studies relied on maternal recall or described coverage rather than timeliness. Suggesting that delay rates in the country may be substantially higher than reported previously if the timeliness is rigorously assessed.

When analysing the visit-specific patterns variation has been observed across the immunization schedule. Early visits (birth till 6 months) had high rates of timely vaccination (ranging from 67.3% to 98.6%), the adherence declined with the increasing age. The highest rate of delayed vaccinations was observed at 9-month age (44.0%), reflecting a period when caregivers show reduced urgency for vaccination as children grow older. The second year showed dramatic increase in missed vaccination (37.5% at 12-month, 36.3% at 18-month visit) along with a sharp decline in timely vaccination (36.3%). This pattern suggests competing priorities, reduced perceived risk of vaccine-preventable diseases by caregivers, or difficulties accessing healthcare facilities that may contribute to declining adherence. These trends have been observed in other LMICs [24].

In this study although the overall coverage rates of the vaccine in the first nine months were high, timeliness of vaccination was suboptimal across all visits. At the 2-month visit, although the coverage was 99.4%, only 73.8% received timely vaccination, leaving a quarter of children unprotected during the first two months of life. This pattern persisted at the 4 and 6-months, 31.0% and 32.1% respectively. The most concerning gaps were observed for the measles vaccine (9-month visit), while overall coverage was high (96.4%), nearly half of the children remained susceptible to measles during a critical period of vulnerability because of delay [25]. This finding is alarming given the recent resurgence of measles outbreaks globally and in the Eastern Mediterranean region. Recent data indicate that Iraq has experienced a resurgence of measles outbreaks in 2023–2024, exacerbated by immunity gaps and service disruptions [26]. The highest coverage gaps were identified at the 12-month and 18-month visits, where 25.6% and 37.5% of children, respectively, missed their scheduled vaccinations. This suggests that in the second year of life once children fall behind schedule, completing the remaining doses becomes increasingly difficult. The gap between coverage and timeliness highlights the hidden vulnerability in Iraq's immunization system that children remain unprotected during critical periods of susceptibility despite eventually receiving vaccines. These findings highlights the need for interventions not only to address early delays but also to target the critical 12–18-month period, where immunization dropout rates are highest [22].

Lack of knowledge related to vaccination schedule was reported by over half of caregivers (54.8%), aligning with previous study in Iraq, where inadequate awareness of parents was identified as a major barrier to childhood immunization [27]. This finding suggest that healthcare providers may not be adequately communicating the timing and importance of follow-up visits. In this study the other reasons of immunization delays (i.e., family opposition to vaccination 19.0%, negative vaccine perception 17.9%, and concerns about vaccine related ADRs 16.1%) highlights the need of health education and family engagement to address the knowledge gaps and barriers due to attitude [1]. Surprisingly vaccine price was identified as a barrier despite vaccines being provided free through the Iraqi EPI, suggesting that indirect costs (transportation and time off work) may still affect timely vaccination [13].

There was a significant association of lack of knowledge of vaccination schedule and perceived vaccine availability with immunization delay. Caregivers not aware of the vaccination schedule had nearly four times higher odds of delayed vaccination (aOR = 3.85; 95% CI: 1.04–14.24), this finding is consistent with studies from other LMICs, where maternal knowledge was a strong predictor of vaccination completion [28-30]. The persistence of this association after controlling education shows that vaccination knowledge is a distinct modifiable factor that can be improved through targeted health education. Caregivers who reported no issues with availability of vaccine had 4.74 times higher odds of delayed vaccination compared to those who didn't cite availability as barrier (aOR = 4.74; 95% CI: 1.09–20.51). This counter intuitive find can be due to several reasons. First, caregivers who faced stock-out became more vigilant or motivated to vaccinate once vaccines are available, whereas those who never faced shortage may be more complacent[31]. Second, caregivers with vaccination delay may not perceive availability as an issue, as they are not seeking vaccination at the scheduled times [32]. Third, there might be reverse causality, where caregivers who are delayed are less likely to encounter stock-out because they are not visiting health centres during peak vaccination period [32]. Fourth, there can be other confounding factors, such as socioeconomic status or health-seeking behaviour influencing both perceived availability and delay, also suggested by wide confidence interval [33]. Further qualitative research is needed to explore the relationship and behaviour pathways of this association.

Notably, none of the sociodemographic factors showed a significant association with immunization delay, this finding is striking as previous studies reported associations between parental education and vaccination outcomes [24]. The absence of such associations may reflect that people living in urban Baghdad, have almost similar access to information and healthcare services demographic subgroups, or vaccination status is mediated by knowledge and access of vaccination. This finding suggests that

strategies focused on caregiver knowledge and vaccine availability may be effective across diverse populations without specific demographic targets.

Based on the key findings, the following recommendations are proposed to address delay of childhood immunization among children in Baghdad, Iraq:

- Strengthen health education efforts for the care-givers especially at the time of first visit. This health education could accompany printed schedules, electronic reminders, and reinforcing key messages related to perception during vaccination visits.
- Ensuring consistent vaccine supply and communicating availability to caregivers to build trust and encouraging timely visits.

Q14: Try to write recomendations based on study findings.

- Targeted outreach, such as reminder calls or home visits, to reduce dropouts.
- Community-based interventions engaging parents, grandparents, and other influential family members to address vaccine hesitancy at the household level.
- Future qualitative studies to explore the reasons behind knowledge gaps and the complex relationship between perceived vaccine availability and delay.
- Targeted catch-up vaccination campaigns for children aged 12–24 months, including dedicated vaccination days, home visits, or mobile vaccination units to reach families who have fallen behind schedule.

This study has several notable strengths. Immunization status was assessed using immunization cards to ensure data collection and minimizing recall bias. The enrolment of children aged 18–24 months allowed complete assessment of all seven essential immunization visits [6, 34]. Finally, the study was conducted in Baghdad, urban capital of Iraq, providing insights relevant to a major metropolitan setting in a post-conflict context [12]. Despite these strengths, this study possess several limitations. Because of the cross-sectional design, the findings only reflects correlations at one point in time and do not provide causality. The use of purposive sampling may limit generalizability to larger population of Baghdad. Post-hoc analysis showed the study had adequate power (≥80%) to detect the large effects for knowledge (OR = 3.85) and vaccine availability (OR = 4.74), the study however had limited power to detect smaller effect sizes (OR < 2.5), and non-significant sociodemographic findings should be interpreted with this limitation in mind. Reported reasons of delays by caregiver may be subjected to social desirability bias. Additionally, the study does not assess factor related to healthcare providers (e.g., information given by healthcare providers, operating hours of vaccination centre, provider attitudes) that may influence timeliness of vaccination.

Conclusion

This study demonstrates that immunization delays are highly prevalent among children in Baghdad, Iraq, and most children experienced delays at multiple visits with a reasonable proportion of children missing doses of vaccines at later visits. Lack of knowledge related to vaccination schedule and perception about vaccine availability were the key modifiable factors associated with vaccination delay. Notably, no sociodemographic characteristics were observed as significant predictors of delays, suggesting interventions focusing on knowledge improvement and smooth supply of vaccines may be effective across diverse populations. As Iraq is continuing to rebuild its healthcare system after decades of internal and external conflicts, strengthening immunization services through targeted education, access to vaccination, and community/family engagement will be essential to achieving high vaccination coverage to reduce the burden of vaccine-preventable diseases among children.

Author contributions

Syed Shaukat Ali Muttqi Shah: Conceptualization, Data curation, Formal analysis, Investigation, Methodology, Writing – original draft, Writing – review & editing

Hakim Ahmed Mukhlif: Investigation, Data curation, Writing – review & editing
Mohammed Abdul Wahab Alwardi: Investigation, Data curation, Methodology, Writing – review & editing
Ale Zehra: Formal analysis, Validation, Writing – review & editing
Uroosa Kanwal: Literature review, Software, Writing – original draft, Writing – review & editing
Tehreem Ansari: Formal analysis, Writing – original draft, Writing – review & editing

Competing Interests

The authors have declared that no competing interests exist.

Funding

This research received no specific grant from any funding agency in the public, commercial, or not-for-profit sectors.

Acknowledgments

The authors thank the staff of the 11 primary healthcare centers in Baghdad for their cooperation and support during data collection. We also thank the caregivers who participated in this study. Special thanks to Dr. Asif Khaliq for his valuable assistance with data analysis.

Data Availability Statement

The anonymized dataset supporting the findings of this study is available as Supporting Information (S4 File). The data have been anonymized to protect participant confidentiality. The original data are available from the corresponding author upon reasonable request.

References:

Q15: Check the referencing type and maintain uniformity

1. Al-Qerem W, Jarab A, Hammad A, Alasmari F, Ling J, Alsajri AH, et al. Iraqi Parents' Knowledge, Attitudes, and Practices towards Vaccinating Their Children: A Cross-Sectional Study. Vaccines. 2022;10(5):820.
2. Al-Mosawi AJ. Iraq healthcare system: An update. Lupine Online Journal of Medical Sciences. 2020;4(3):404-11. https://doi.org/10.32474/LOJMS.2020.04.000190.
3. Nandi A, Shet A. Why vaccines matter: understanding the broader health, economic, and child development benefits of routine vaccination. Human vaccines & immunotherapeutics. 2020;16(8):1900-4. https://doi.org/10.1080/21645515.2019.1708669.
4. Shattock AJ, Johnson HC, Sim SY, Carter A, Lambach P, Hutubessy RC, et al. Contribution of vaccination to improved survival and health: modelling 50 years of the Expanded Programme on Immunization. The Lancet. 2024;403(10441):2307-16. https://doi.org/ 10.1016/S0140-6736(24)00850-X
5. Mirza I, Lemango ET, Lindstrand A. Expanded Programme on Immunization (EPI): a legacy of 50 years and the road ahead. MDPI; 2025. p. 649.
6. Molan JAS. Factors influencing compliance with immunization regimen among mother in Basrah, Iraq. The Medical Journal of Basrah University. 2020;38(1):37-46. https://doi.org/10.33762/mjbu.2020.126642.1002.
7. Hussein RG. Assessment of Routine Vaccination Coverage among Selected Sample of Children less Than Five Years. 2020. https://doi.org/10.26505/DJM.19025590913.
8. Modlin JF, Schaffner W, Orenstein W, Bandyopadhyay AS. Triumphs of immunization. The Journal of Infectious Diseases. 2021;224(Supplement_4):S307-S8. https://doi.org/10.1093/infdis/jiab123.

9. Muhoza P, Shah MP, Amponsa-Achiano K, Gao H, Quaye P, Opare W, et al. Timeliness of childhood vaccinations following strengthening of the second year of life (2YL) immunization platform and introduction of catch-up vaccination policy in Ghana. Vaccines. 2024;12(7):716. https://doi.org/10.3390/vaccines12070716.
10. Raguindin PF, Morales-Dizon M, Aldaba J, Mangulabnan LP, Reyes RP, Batmunkh N, et al. Timeliness of childhood vaccinations in the Philippines. Journal of Public Health Policy. 2021;42(1):53-70. https://doi.org/10.1057/s41271-020-00255-w.
11. Jadoo SAA, Alhusseiny AH, Yaseen SM, Al-Samarrai MAM, Mahmood AS. Evaluation of health system in Iraq from people's point of view: a comparative study of two different eras. Journal of Ideas in Health. 2021;4(2):380-8. https://doi.org/10.47108/jidhealth.Vol4.Iss2.100.
12. Lafta RK, Al-Nuaimi MA. War or health: a four-decade armed conflict in Iraq. Medicine, Conflict and Survival. 2019;35(3):209-26. https://doi.org/10.1080/13623699.2019.1670431.
13. MoH. Annual staticstics report 2023. Ministry of Health, Republic of Iraq; 2023.
14. Aldajani FN, Aldosari M. Pharmacist-led vaccination services in the Middle East. Journal of Pharmaceutical Policy and Practice. 2023;16(1):171. https://doi.org/10.1186/s40545-023-00664-8.
15. Syed Alwi S, Rafidah E, Zurraini A, Juslina O, Brohi IB, Lukas S. A survey on COVID-19 vaccine acceptance and concern among Malaysians. BMC public health. 2021;21(1):1129.
16. MOH. National vaccination schedule: Republic of Iraq, Iraqi Ministry of Health; 2021 [updated 16/09/2021. Childhood vaccination schedule]. Available from: https://moh.gov.iq/?page=36.
17. Al-lela OQB, Bahari mb, Baderden S, Basher A, Hamoodi H. Factors Affecting on Immunization Compliance: Iraq. Journal of Pharmacy Practice and Community Medicine. 2017;3:246-53. https://doi.org/10.5530/jppcm.2017.4.67.
18. Alhaddad AR, Ahmadnezhad E, Fotouhi A. The vaccination coverage rate in under-5 children in Nasiriyah, Iraq before and during the COVID-19 pandemic. Epidemiology and Health. 2022;44:e2022035. https://doi.org/10.4178/epih.e2022035.
19. Khaliq A, Sayed SA, Hussaini SA, Azam K, Qamar M. Missed Immunization Opportunities Among Children Under 5 Years Of Age Dwelling In Karachi City. J Ayub Med Coll Abbottabad. 2017;29(4):645-9.
20. Headley TY, Shay CW, Tozan Y. The impact of armed conflict on vaccination coverage: a systematic review of empirical evidence from 1985 to 2025. Conflict and Health. 2025;19(1):71. https://doi.org/10.1186/s13031-025-00708-7.
21. Tippins A, Leidner AJ, Meghani M, Griffin A, Helgenberger L, Nyaku M, Underwood JM. Timeliness of childhood vaccination in the Federated States of Micronesia. Vaccine. 2017;35(47):6404-11. https://doi.org/10.1016/j.vaccine.2017.10.001.
22. Obaid AF, Abdulrasol ZA, Shlash AMJ, Tuman MR, Hussain MD. Assessment of missing opportunity of vaccination at primary health care center: A retrospective study. J Contemp Med Sci| Vol. 2023;9(1):77-81. https://doi.org/10.22317/jcms.v9i1.1321.
23. Essa HS, Abdulrahem DM, Waheeb HN, editors. Overview of Immunization Coverage and Determinants for Children. Medical Forum Monthly; 2025.https://doi.org/10.60110/medforum.360303.
24. Budu E, Darteh EKM, Ahinkorah BO, Seidu A-A, Dickson KS. Trend and determinants of complete vaccination coverage among children aged 12-23 months in Ghana: Analysis of data from the 1998 to 2014 Ghana Demographic and Health Surveys. PLOS ONE. 2020;15(10):e0239754. https://doi.org/10.1371/journal.pone.0239754.
25. Wariri O, Okomo U, Kwarshak YK, Utazi CE, Murray K, Grundy C, Kampmann B. Timeliness of routine childhood vaccination in 103 low-and middle-income countries, 1978–2021: A scoping review to map measurement and methodological gaps. PLOS global public health. 2022;2(7):e0000325. https://doi.org/10.1371/journal.pgph.0000325.

26. Al-Tawfiq JA, Memish ZA. Measles outbreaks in the Eastern Mediterranean Region: urgent need for strengthened vaccination efforts. Journal of Epidemiology and Global Health. 2024;14(2):252-4. https://doi.org/10.1007/s44197-024-00227-3.
27. Al-lela OQB, Bahari MB, Salih MRM, Al-abbassi MG, Elkalmi RM, Jamshed SQ. Factors underlying inadequate parents' awareness regarding pediatrics immunization: findings of cross-sectional study in Mosul- Iraq. BMC Pediatrics. 2014;14(1):29. https://doi.org/10.1186/1471-2431-14-29.
28. Oyato BT, Abasimel HZ, Lama T, Hussein D, Medina S. Vaccination delay and associated factors among children of age 12–23 months in Gomma district, Oromia, Ethiopia, 2022. Scientific reports. 2025;15(1):18461. https://doi.org/10.1038/s41598-025-97882-8.
29. Nour TY, Farah AM, Ali OM, Osman MO, Aden MA, Abate KH. Predictors of immunization coverage among 12–23 month old children in Ethiopia: systematic review and meta-analysis. BMC public health. 2020;20(1):1803. https://doi.org/10.1186/s12889-020-09890-0.
30. Dejene H, Girma D, Geleta LA, Legesse E. Vaccination timeliness and associated factors among children aged 12–23 months in Debre Libanos district of North Shewa Zone, Oromia Regional State, Ethiopia. Frontiers in Pediatrics. 2022;10:867846. https://doi.org/10.3389/fped.2022.867846.
31. Walekhwa AW, Musoke D, Nalugya A, Biribawa C, Nsereko G, Wafula ST, et al. Gaps in measles vaccination coverage in Kasese district, Western Uganda: results of a qualitative evaluation. BMC infectious diseases. 2022;22(1):589. https://doi.org/10.1186/s12879-022-07579-w.
32. Mkamba BS, Rutungwa E, Karimi PN, Ngenzi JL. Factors that influence the availability of childhood vaccine in healthcare facilities at Tana River County, Kenya. Journal of Pharmaceutical Policy and Practice. 2023;16(1):142. https://doi.org/10.1186/s40545-023-00648-8.
33. Feldstein LR, Sutton R, Jalloh MF, Parmley L, Lahuerta M, Akinjeji A, et al. Access, demand, and utilization of childhood immunization services: A cross-sectional household survey in Western Area Urban district, Sierra Leone, 2019. Journal of global health. 2020;10(1):010420. https://doi.org/10.7189/jogh.10.010420.
34. Mohammed SI, Sabry AT, Sabry DT, Mohammed BH. Knowledge and malpractices in pediatrics diarrhea management by Iraqi mothers. Asian Journal of Pharmaceutical and Clinical Research. 2018;11(8):503-7. https://doi.org/10.22159/ajpcr.2018.v11i8.27454.

Supplementary files

S1 File. Study Questionnaire. The pre-validated structured questionnaire used for data collection (adopted and adapted from Khaliq et al., 2017)
S2 File. Anonymized Dataset. Anonymized data in Excel format.
S3 File. STROBE Checklist. Completed STROBE checklist for cross-sectional studies.
S4 File. Data Dictionary. Variable definitions and coding scheme.

Ethical approval

Click here to access/download

Supporting Information

irb vaccination.pdf

S1 File

Click here to access/download
Supporting Information
S1 file_Questionnaire.docx

S2 File

Click here to access/download

Supporting Information

S2 file_Data Dictionary.docx

Click here to access/download
Supporting Information
S3 file_STROBEChecklist.docx

S4 File

Click here to access/download

Supporting Information

S4 file_Anonimyzed dataset.xlsx

Click here to access/download
Supporting Information - Compressed/ZIP File Archive
PLOSOne_Human_Subjects_Research_Checklist.docx
