# Supplementary material for: Immunization timeliness and its determinants among young children in Baghdad, Iraq: A cross-sectional study using documented vaccination cards
Source: PLoS One. 2026 Oct 1;21(10):e0359962. doi: 10.1371/journal.pone.0359962 (PMC13630201; doi:10.1371/journal.pone.0359962)
Supplement: Supplementary file 2 [file pone.0359962.s001.docx]

## Questionnaire

Exploring the knowledge and compliance of Immunization among women bearing a child under Two Years of Age in Iraq

**SECTION A: VISIT INFORMATION:**

1. Cluster code: ___ ___
2. Screening number: ___ ___ ___ ___
3. Date of screening: /___ ___/___ ___/___ ___/
4. Consented to provide basic information:

- Yes
- No

**SECTION B: SOCIO-DEMOGRAPHIC INFORMATION:**

Take the following information about the family of the participant?

| **S.No** | **Reasons** | **Number** |
| --- | --- | --- |
| 5a | What is mother age? | **___ ___** |
| 5b | How many year of education mother has received? | **___ ___** |
| 5c | What is the age of father? | **___ ___** |
| 5d | How many year of education father has received? | **___ ___** |
| 5e | How many children mother has delivered? (including still birth) | **___ ___** |
| 5f | How many children mother has delivered? (live births) | **___ ___** |
| 5g | How many children of your own family die after birth? | **___ ___** |
| 5h | What is the child order of birth? (1^st^ baby, 2^nd^ baby, 3^rd^ baby etc) | ___ ___ |
| 5i | How many people in your family live in a house? | **___ ___** |

**SECTION C: DETEMINATION OF ELIGIBILITY:**

| **S.No** | **Reasons** | **YYes** | **NNo** |
| --- | --- | --- | --- |
| 6a | Do you have any child aged less than 2 year? |  |  |
| 6b | Is primary caregiver of that child available? |  |  |
| 6c | Is that child alive? |  |  |
| 6d | Do you have vaccination card of your children? |  |  |
| 6e | Are you living permanently in Baghdad since last 2 year? |  |  |
| 6f | Is the primary caregiver able to understand local language Arabic? |  |  |
| 6g | Are you providing us the vaccination card of your children for review? |  |  |
| 6h | Are you providing us information regarding your child vaccination? |  |  |

**7.** Is the participant eligible to participate (If all answer of the above section are “YES” then the child would be considered as eligible)?

- Yes
- No (If No write reason for exclusion ineligibility)

8. The participant is not eligible because:

- No under 2 year children at home
- Child was not alive
- Primary caregiver is not present
- Primary caregiver was unable to understand local language Arabic
- Guest or visitors in study area
- Family do not have vaccination card
- Family refused to participate

9. Participant Identification Number: ___ ___ ___

**SECTION D: IMMUNIZATION IMPORTANCE INFORMATION:**

9. On scale of 1 to 5, where 1 is not favor at all, 5 is favor and 3 is the midpoint Are you in favor of vaccination?

|  | 1 | 2 | 3 | 4 | 5 |
| --- | --- | --- | --- | --- | --- |
| Are you in Favour of Vaccination |  |  |  |  |  |

10. On scale of 1 to 5, where 1 is not familiar at all, 5 is familiar and 3 is the midpoint. To what extent are you familiar with recommended childhood immunization? ( i.e. which short is given when and for what?)

|  | 1 | 2 | 3 | 4 | 5 |
| --- | --- | --- | --- | --- | --- |
| Familiar with childhood immunization |  |  |  |  |  |

11. Do you know how many visits in health care facility for child immunization is needed during first year of life?

- Yes
- No

11a. If Yes, please specify: ______________________

12. Do you know how many visits in health care facility for child immunization is needed during second year of life?

- Yes
- No

12a. If Yes, please specify: _____________

19. In your opinion breast feeding is essential from the babies growth and immunity;
    - Yes
    - No
20. Did you breast feed your child?
    a. Yes
    b. No

If yes,

20a. How long you did exclusive breast feeding of your child _________ months

20b. At what age your started giving other food in addition to the breast milk ________ months

20c. At what age you stopped the breast feeding of your child ___________

If no, why you didn’t do the breast feeding of your child (write reasons)

_____________________________________________________________________

**SECTION E: VACCINATION STATUS:**

13. Do you vaccinate your children against vaccine preventable diseases?

- Yes
- No

14. What is the date of birth of child? /__ __ / __ __ / __ __ __ __/
15. What is the child sex? __________________
16. What is the birth place of this child?

- Home
- Maternity clinic
- Hospital

17. With the help of vaccination card, compare the date of vaccine administration with the schedule vaccination date and decide that either the child have received vaccination either on time, delayed, missed or invalid

^(Write “T” if the vaccine is administered within the window period or on time, “I” if the vaccine was administered 5 days before the schedule date, “D” if the vaccine was administered not on schedule date, “M” if the vaccine was not administered)^

| **Time of Vaccination** | **Vaccine Name** | **Schedule Date** | **Administration Date** | **Status** |
| --- | --- | --- | --- | --- |
| At Birth | Hepatitis B |  |  |  |
| 1 week | OPV |  |  |  |
|  | BCG |  |  |  |
| 2 months | OPV-I |  |  |  |
|  | Penta-I |  |  |  |
|  | Pneumo-I |  |  |  |
|  | Rotavirus – I |  |  |  |
| 4 months | OPV-II |  |  |  |
|  | Penta-II |  |  |  |
|  | IPV-I |  |  |  |
|  | Pneumo-II |  |  |  |
|  | Rotavirus – II |  |  |  |
| 6 months | OPV-III |  |  |  |
|  | Penta-III |  |  |  |
|  | IPV-II |  |  |  |
|  | Pneumo-III |  |  |  |
|  | Rotavirus - III |  |  |  |
| 9 months | Measles-I |  |  |  |
|  | Vitamin A 10K |  |  |  |
| 12 months | Multi Measles I |  |  |  |
| 18 months | OPV Boaster I |  |  |  |
|  | Triple vac. DPT - I |  |  |  |
|  | Multi Measles – II |  |  |  |
|  | Vitamin A 20K |  |  |  |
| 4-6 years | OPV Boaster II |  |  |  |
|  | Triple vac, DPT II |  |  |  |
|  | Vitamin A 20K |  |  |  |

**SECTION F: REASONS FOR DELAYED, INVALID AND MISSED IMMUNIZATION:**

Skip this section if the child has received all vaccines on schedule dates

| **S.No** | **Reasons** | **Yes** | **No** |
| --- | --- | --- | --- |
| 18a | No vaccination center in the catchment area |  |  |
| 18b | Family decision makers are against vaccination |  |  |
| 18c | Unavailability of vaccine in the vaccination center |  |  |
| 18d | Migration of the family |  |  |
| 18e | Cost of the vaccine was not covered by government |  |  |
| 18f | Vaccination cause serious side effects |  |  |
| 18g | Child was too sick |  |  |
| 18h | Doctor said that it was not safe to vaccinate |  |  |
| 18i | Did not know about the vaccination schedule |  |  |
| 18j | Don’t trust the government |  |  |
| 18k | Too many needles/injections, it traumatizes the child |  |  |
|  | Others. (specify) |  |  |

**SECTION G: DATA QUALITY CONTROL AND ASSURANCE**

19. ID of person completing the form: ________________	Dated: ___/ ___/ ____

20. ID of person reviewing the form: ________________	Dated: ___/ ___/ ____

21. ID of person entering the form: ________________	Dated: ___/ ___/ ____
