# Supplementary material for: Immunization timeliness and its determinants among young children in Baghdad, Iraq: A cross-sectional study using documented vaccination cards
Source: PLoS One. 2026 Oct 1;21(10):e0359962. doi: 10.1371/journal.pone.0359962 (PMC13630201; doi:10.1371/journal.pone.0359962)
Supplement: Supplementary file 3 [file pone.0359962.s002.docx]

**Supplementary File S2: Data Dictionary**

**Study: Exploring the Determinants of Pediatric Immunization Status Among Young Children in Baghdad, Iraq**

| Variable Name | Label | Values/Coding | Type | Description |
| --- | --- | --- | --- | --- |
| **Demographic Variables** |  |  |  |  |
| Q5a.MotherAge | Mother's age | 1 = Below 20 years; 2 = 20–29 years; 3 = 30–39 years; 4 = 40–49 years | Categorical | Age of the mother |
| Q5b.MotherEducation | Mother's education | 1 = No education; 2 = Primary; 3 = Secondary; 4 = Higher | Categorical | Highest level of education completed by mother |
| Q5c.FatherAge | Father's age | 1 = Below 20 years; 2 = 20–29 years; 3 = 30–39 years; 4 = 40–49 years | Categorical | Age of the father |
| Q5d.FatherEducation | Father's education | 1 = No education; 2 = Primary; 3 = Secondary; 4 = Higher | Categorical | Highest level of education completed by father |
| Q5h.Order.of.child.birth | Birth order | 1 = Index child (first); 2 = Subsequent child | Categorical | Birth order of the child |
| Q5i.Family.Size | Family size | 1 = Small (≤5 members); 2 = Medium/Large (>5 members) | Categorical | Number of family members |
| Q15.SexOfChild | Child's sex | 1 = Male; 2 = Female | Categorical | Sex of the child |
| Q16.Birthplace | Place of delivery | 1 = Home delivery; 2 = Institutional delivery | Categorical | Where the child was born |
| **Vaccination Status Variables** |  |  |  |  |
| Week_1 | 1st week vaccination | 1 = Timely; 2 = Invalid; 3 = Delayed; 4 = Missed | Categorical | Status of birth dose vaccination |
| Month_2 | 2-month vaccination | 1 = Timely; 2 = Invalid; 3 = Delayed; 4 = Missed | Categorical | Status of 2-month vaccination |
| Month_4 | 4-month vaccination | 1 = Timely; 2 = Invalid; 3 = Delayed; 4 = Missed | Categorical | Status of 4-month vaccination |
| Month_6 | 6-month vaccination | 1 = Timely; 2 = Invalid; 3 = Delayed; 4 = Missed | Categorical | Status of 6-month vaccination |
| Month_9 | 9-month vaccination | 1 = Timely; 2 = Invalid; 3 = Delayed; 4 = Missed | Categorical | Status of 9-month vaccination |
| Month_12 | 12-month vaccination | 1 = Timely; 2 = Invalid; 3 = Delayed; 4 = Missed | Categorical | Status of 12-month vaccination |
| Month_18 | 18-month vaccination | 1 = Timely; 2 = Invalid; 3 = Delayed; 4 = Missed | Categorical | Status of 18-month vaccination |
| **Refusal/Barrier Variables** |  |  |  |  |
| Q18b.FamilyaganistVac | Family against vaccination | 0 = No; 1 = Yes | Binary | Family opposition to vaccination |
| Q18c.AvaialbilityVac | Vaccine availability | 0 = Available; 1 = Not available | Binary | Vaccine availability at health center |
| Q18d.Migration | Family migration | 0 = No; 1 = Yes | Binary | Recent family migration |
| Q18e.CostCover | Vaccine cost | 0 = No; 1 = Yes | Binary | Cost as a barrier |
| Q18f.SideEffects | Concerns about side effects | 0 = No; 1 = Yes | Binary | Fear of vaccine side effects |
| Q18g.Sick | Child was sick | 0 = No; 1 = Yes | Binary | Child illness at scheduled visit |
| Q18h.VacNotSafe | Vaccine not safe | 0 = No; 1 = Yes | Binary | Perception that vaccine is unsafe |
| Q18i.VacSchedule | Knowledge of schedule | 0 = Knows; 1 = Does not know | Binary | Caregiver knowledge of vaccination schedule |
| Q18j.GovtTrust | Trust in government | 0 = Trust; 1 = Does not trust | Binary | Trust in government policies |
| Q18k.Fear | Fear of vaccination | 0 = No; 1 = Yes | Binary | Fear regarding vaccination |
| **Computed Variables** |  |  |  |  |
| timely_count | Count of timely doses | 0–7 | Continuous | Number of timely doses received |
| nontimely_count | Count of non-timely doses | 0–7 | Continuous | Number of delayed, invalid, or missed doses |
| delay_binary | Immunization status (binary) | 0 = Timely; 1 = Delayed | Binary | Overall immunization status |
| vaccination_status | Immunization status (3 categories) | 1 = Timely; 2 = One delay; 3 = Two or more delays | Categorical | Overall immunization status with delay severity |
| delay_count | Count of delayed/missed doses | 0–7 | Continuous | Number of doses delayed or missed |
| delay_category | Immunization delay severity | 1 = Timely; 2 = One delay; 3 = Two delays; 4 = Three or more delays | Categorical | Severity of immunization delay |
| Mother_Education_Group | Mother's education (grouped) | 1 = No education/Primary; 2 = Secondary; 3 = Higher | Categorical | Grouped maternal education |
| Father_Education_Group | Father's education (grouped) | 1 = No education/Primary; 2 = Secondary; 3 = Higher | Categorical | Grouped paternal education |
| barrier_count | Composite barrier score | 0–7 | Continuous | Sum of barriers reported by caregiver |
