# Supplementary material for: Immunization timeliness and its determinants among young children in Baghdad, Iraq: A cross-sectional study using documented vaccination cards
Source: PLoS One. 2026 Oct 1;21(10):e0359962. doi: 10.1371/journal.pone.0359962 (PMC13630201; doi:10.1371/journal.pone.0359962)
Supplement: Supplementary file 4 [file pone.0359962.s003.docx]

**STROBE Statement – Checklist for Cross-Sectional Studies**

**Manuscript Title:** Immunization Timeliness and Its Determinants Among Young Children in Baghdad, Iraq: A Cross-Sectional Study Using Documented Vaccination Cards

| Section/Item | Item No | Recommendation | Reported on Page/Line |
| --- | --- | --- | --- |
| **Title and abstract** |  |  |  |
| Title | 1a | Indicate the study's design with a commonly used term in the title | Page 1 |
| Abstract | 1b | Provide in the abstract an informative and balanced summary of what was done and what was found | Page 1 |
| **Introduction** |  |  |  |
| Background/rationale | 2 | Explain the scientific background and rationale for the investigation being reported | Pages 2 |
| Objectives | 3 | State specific objectives, including any prespecified hypotheses | Line 70-82 |
| **Methods** |  |  |  |
| Study design | 4 | Present key elements of study design early in the paper | Line 84-89 |
| Setting | 5 | Describe the setting, locations, and relevant dates | Line 84-89 |
| Participants | 6 | (a) Give the eligibility criteria, and the sources and methods of selection of participants | Line 90-104 |
| Variables | 7 | Clearly define all outcomes, exposures, predictors, potential confounders, and effect modifiers | Line 129-152 |
| Data sources/measurement | 8 | For each variable of interest, give sources of data and details of methods of assessment | Pages 3-4 |
| Bias | 9 | Describe any efforts to address potential sources of bias | Line 152-162, 392-405 |
| Study size | 10 | Explain how the study size was arrived at | Line 109-119 |
| Quantitative variables | 11 | Explain how quantitative variables were handled in the analyses | Line 129-152 |
| Statistical methods | 12 | (a) Describe all statistical methods, including those used to control for confounding | Line 163-174 |
|  |  | (b) Describe any methods used to examine subgroups and interactions | N/A |
|  |  | (c) Explain how missing data were addressed | Line 174-176 |
|  |  | (d) Describe any sensitivity analyses | NA |
| **Results** |  |  |  |
| Participants | 13 | (a) Report numbers of individuals at each stage of study | 192-195 and Figure 1 |
|  |  | (b) Give reasons for non-participation at each stage | 192-195 and Figure 1 |
| Descriptive data | 14 | (a) Give characteristics of study participants | Pages 6; Table 1 |
|  |  | (b) Indicate number of participants with missing data | Page 6 |
| Outcome data | 15 | Report numbers of outcome events or summary measures | Pages 6-9; Figures 2-5 |
| Main results | 16 | (a) Give unadjusted estimates and, if applicable, confounder-adjusted estimates | Table 2 |
|  |  | (b) Report category boundaries when continuous variables were categorized | Table 1 |
|  |  | (c) If relevant, consider translating estimates of relative risk into absolute risk | N/A |
| Other analyses | 17 | Report other analyses done—e.g., analyses of subgroups and interactions | N/A |
| **Discussion** |  |  |  |
| Key results | 18 | Summarize key results with reference to study objectives | Page 13 |
| Limitations | 19 | Discuss limitations of the study, taking into account sources of potential bias | Page 13 |
| Interpretation | 20 | Give a cautious overall interpretation of results considering objectives, limitations, and multiplicity of analyses | Pages 11-13 |
| Generalizability | 21 | Discuss the generalizability (external validity) of the study results | Page 11-13 |
| **Other information** |  |  |  |
| Funding | 22 | Give the source of funding and the role of the funders | Page 14 |
