# Supplementary material for: Immunization timeliness and its determinants among young children in Baghdad, Iraq: A cross-sectional study using documented vaccination cards
Source: PLoS One. 2026 Oct 1;21(10):e0359962. doi: 10.1371/journal.pone.0359962 (PMC13630201; doi:10.1371/journal.pone.0359962)
Supplement: Peer review history

# Peer review: Decision Letter 0

**DOI:** 10.1371/journal.pone.0359962.r001

**Authors:** Mohammed Badeso

2 Sep 2026

Dear Dr. Shah,

Thank you for submitting your manuscript to PLOS One. After careful consideration, we feel that it has merit but does not fully meet PLOS One’s publication criteria as it currently stands. Therefore, we invite you to submit a revised version of the manuscript that addresses the points raised during the review process.

Please submit your revised manuscript by Oct 17 2026 11:59PM. If you will need more time than this to complete your revisions, please reply to this message or contact the journal office at plosone@plos.org. When you're ready to submit your revision, log on to https://www.editorialmanager.com/pone/ and select the 'Submissions Needing Revision' folder to locate your manuscript file.

- A letter that responds to each point raised by the academic editor and reviewer(s). You should upload this letter as a separate file labeled 'Response to Reviewers'.
- A marked-up copy of your manuscript that highlights changes made to the original version. You should upload this as a separate file labeled 'Revised Manuscript with Track Changes'.
- An unmarked version of your revised paper without tracked changes. You should upload this as a separate file labeled 'Manuscript'.

If you would like to make changes to your financial disclosure, please include your updated statement in your cover letter. Guidelines for resubmitting your figure files are available below the reviewer comments at the end of this letter.

If applicable, we recommend that you deposit your laboratory protocols in protocols.io to enhance the reproducibility of your results. Protocols.io assigns your protocol its own identifier (DOI) so that it can be cited independently in the future. For instructions see: https://journals.plos.org/plosone/s/submission-guidelines#loc-laboratory-protocols. Additionally, PLOS One offers an option for publishing peer-reviewed Lab Protocol articles, which describe protocols hosted on protocols.io. Read more information on sharing protocols at https://plos.org/protocols?utm_medium=editorial-email&utm_source=authorletters&utm_campaign=protocols.

As the corresponding author, your ORCID iD is verified in the submission system and will appear in the published article. PLOS supports the use of ORCID, and we encourage all coauthors to register for an ORCID iD and use it as well. Please encourage your coauthors to verify their ORCID iD within the submission system before final acceptance, as unverified ORCID iDs will not appear in the published article. *Only*  the individual author can complete the verification step; PLOS staff *cannot*  verify ORCID iDs on behalf of authors.

We look forward to receiving your revised manuscript.

Kind regards,

Mohammed Hasen Badeso, Epidemiologist

Academic Editor

PLOS One

Journal Requirements:

When submitting your revision, we need you to address these additional requirements.

1. Please ensure that your manuscript meets PLOS ONE's style requirements, including those for file naming. The PLOS ONE style templates can be found at

https://journals.plos.org/plosone/s/file?id=wjVg/PLOSOne_formatting_sample_main_body.pdf and

https://journals.plos.org/plosone/s/file?id=ba62/PLOSOne_formatting_sample_title_authors_affiliations.pdf

2. In the online submission form, you indicated that “The original data are available from the corresponding author upon reasonable request.”

All PLOS journals now require all data underlying the findings described in their manuscript to be freely available to other researchers, either 1. In a public repository, 2. Within the manuscript itself, or 3. Uploaded as supplementary information.

This policy applies to all data except where public deposition would breach compliance with the protocol approved by your research ethics board. If your data cannot be made publicly available for ethical or legal reasons (e.g., public availability would compromise patient privacy), please explain your reasons on resubmission and your exemption request will be escalated for approval.

3. We note you have included a table to which you do not refer in the text of your manuscript. Please ensure that you refer to Table 1 in your text; if accepted, production will need this reference to link the reader to the Table.

4. If the reviewer comments include a recommendation to cite specific previously published works, please review and evaluate these publications to determine whether they are relevant and should be cited. There is no requirement to cite these works unless the editor has indicated otherwise.

[Note: HTML markup is below. Please do not edit.]

Reviewers' comments:

Reviewer's Responses to Questions

**Comments to the Author**

1. Is the manuscript technically sound, and do the data support the conclusions?

Reviewer #1: Partly

Reviewer #2: Yes

**********

2. Has the statistical analysis been performed appropriately and rigorously?

Reviewer #1: Yes

Reviewer #2: Yes

**********

3. Have the authors made all data underlying the findings in their manuscript fully available?

The PLOS Data policy

Reviewer #1: Yes

Reviewer #2: Yes

**********

4. Is the manuscript presented in an intelligible fashion and written in standard English?

Reviewer #1: Yes

Reviewer #2: Yes

**********

Reviewer #1: I reviewed the manuscript. Overall, the topic is relevant and the use of vaccination cards is a strength, but several methodological and interpretation issues should be improved before submission/revision.

Main points to improve

1. Major issue: vaccination schedule inconsistency

Introduction says visits are at 1st week, 2, 4, 6, 9, 12 and 18 months, while Methods mention 15 months in one place. This must be corrected and made consistent.

2. Definition of “delay” needs stronger justification

The study defines delay as >28 days after the scheduled date, but the rationale/reference for this 28-day window should be clearly explained.

Clarify whether the 28-day window is based on Iraqi MoH, WHO, or a previous published definition.

3. Sampling limits generalizability

Only 11 of 30 PHCs were purposively selected, and participants were approached by convenience. Therefore, the results should not be presented as representative of Baghdad.

This limitation is mentioned, but should be emphasized more strongly in the Abstract/Conclusion.

4. Statistical analysis needs reconsideration

The sample is only 168, yet many predictors were evaluated.

The use of univariable p-value ≤0.20 followed by backward elimination based on p-values is not ideal and may produce unstable models.

The manuscript should explain the number of events and justify the final model more carefully.

5. Potentially problematic interpretation of vaccine availability

The finding that caregivers reporting no vaccine availability problem had higher odds of delay (aOR 4.74) is counterintuitive.

The Discussion currently gives several speculative explanations. Better to state clearly that this is an unexpected association that may reflect reverse causation, measurement error, residual confounding, or small sample size, rather than suggesting causal mechanisms.

6. Check numerical inconsistencies

There appear to be discrepancies between the reported percentages for missed doses at 12 and 18 months in different sections.

For example, the Results report 63/168 = 37.5% missed at 12 months, but the Discussion later refers to 25.6%. These numbers need careful checking.

7. Discussion is too long and somewhat speculative: Several paragraphs repeat the Results.

Reviewer #2: This is a good manuscript that brings out issues with primary immunization in post conflict zone. It will be a good learning exercise for others who work in similar settings. I have attached a file with 15 questions to be responded.

**********

what does this mean?). If published, this will include your full peer review and any attached files.

If you choose “no”, your identity will remain anonymous but your review may still be made public.

**Do you want your identity to be public for this peer review?**  For information about this choice, including consent withdrawal, please see our Privacy Policy

Reviewer #1: **Yes:**  Mohammad Ashraful Amin

Reviewer #2: **Yes:**  Dr Rahul Narang

**********

[NOTE: If reviewer comments were submitted as an attachment file, they will be attached to this email and accessible via the submission site. Please log into your account, locate the manuscript record, and check for the action link "View Attachments". If this link does not appear, there are no attachment files.]

To ensure your figures meet our technical requirements, please review our figure guidelines: https://journals.plos.org/plosone/s/figures

You may also use PLOS’s free figure tool, NAAS, to help you prepare publication quality figures: https://journals.plos.org/plosone/s/figures#loc-tools-for-figure-preparation.

NAAS will assess whether your figures meet our technical requirements by comparing each figure against our figure specifications.

Attachment Submitted filename: PONE-D-26-19652_reviewer (1).pdf

# Author comment: Author response to Decision Letter 1

**DOI:** 10.1371/journal.pone.0359962.r002

18 Sep 2026

Response to Reviewers

PLOS ONE Manuscript PONE-D-26-19652: "Immunization Timeliness and Its Determinants Among Young Children in Baghdad, Iraq: A Cross-Sectional Study Using Documented Vaccination Cards"

Date: 10, September 2026

Dear Dr. Badeso and Reviewers,

Thank you for the thoughtful and constructive feedback on our manuscript. We have carefully addressed all comments raised by the reviewers and the academic editor. Below we provide a point-by-point response to each comment, with detailed explanations of the changes made and specific line numbers where changes appear in the revised manuscript as tracked changes.

RESPONSE TO REVIEWER #1

Comment 1: "Introduction says visits are at 1st week, 2, 4, 6, 9, 12 and 18 months, while Methods mention 15 months in one place. This must be corrected and made consistent."

Response: We have corrected the Methods section to consistently reflect the seven essential immunization visits as: 1st week, 2 months, 4 months, 6 months, 9 months, 12 months, and 18 months. The reference to 15 months has been removed and replaced with 12 months.

Changes made: Line 113: Changed "15 months" to "12 months"

Comment 2: "Definition of 'delay' needs stronger justification. The study defines delay as >28 days after the scheduled date, but the rationale/reference for this 28-day window should be clearly explained."

Response: We have provided a stronger justification for the 28-day window definition with references to WHO recommendations, published literature, and Iraqi PHC operational practice.

Changes made: Lines 168-172: Added comprehensive justification for 28-day window with references [6, 16, 19, 20]

Comment 3: "Only 11 of 30 PHCs were purposively selected, and participants were approached by convenience. Therefore, the results should not be presented as representative of Baghdad. This limitation is mentioned, but should be emphasized more strongly in the Abstract/Conclusion."

Response: We have strengthened the acknowledgment of this limitation in the Abstract, Discussion, and Conclusion.

Changes made:

• Line 36: Abstract - Added: "However, due to purposive sampling, these findings may not be generalizable to all of Baghdad or Iraq."

• Lines 489-492: Discussion - Strengthened: "The use of purposive sampling, with only 11 of 30 contacted PHCs participating, significantly limits generalizability to the larger population of Baghdad. Therefore, these findings should be considered as indicative of the challenges in urban PHC settings rather than representative of all communities in Baghdad or Iraq."

• Lines 514-5166: Conclusion - Added: "Although these findings provide important insights into vaccination challenges in a major urban post-conflict setting, they should not be generalized to all of Baghdad or Iraq."

Comment 4: "The sample is only 168, yet many predictors were evaluated. The use of univariable p-value ≤0.20 followed by backward elimination based on p-values is not ideal and may produce unstable models. The manuscript should explain the number of events and justify the final model more carefully."

Response: We have revised the Data Analysis section to provide transparent justification with EPV calculation.

Changes made:

• Lines 220-224: Methods - Data Analysis - Added EPV calculation: "With 154 children experiencing delay (the outcome event) and 7 variables in the final model, the events-per-variable ratio was 22:1, exceeding the recommended minimum of 10:1 for logistic regression analysis."

• Lines 496-498: Discussion - Limitations - Added: "The sample size of 168 may limit model stability and the ability to detect smaller but potentially meaningful associations, a larger sample would have allowed for more robust multivariable modelling with additional covariates."

Comment 5: "The finding that caregivers reporting no vaccine availability problem had higher odds of delay (aOR 4.74) is counterintuitive. The Discussion currently gives several speculative explanations. Better to state clearly that this is an unexpected association that may reflect reverse causation, measurement error, residual confounding, or small sample size."

Response: We have revised the Discussion to cautiously interpret this counterintuitive finding, removing speculative causal explanations.

Changes made: Lines 446-451: Discussion - Reframed: "This counter intuitive find should be interpreted with caution. Rather than giving a causal relationship, this unexpected association may reflect reverse causation, measurement error, residual confounding, or small sample size. The wider confidence interval (1.09-20.51) and small number of caregivers who reported availability as barrier (n=21) may further suggest that the finding may be unstable."

Comment 6: "There appear to be discrepancies between the reported percentages for missed doses at 12 and 18 months in different sections. For example, the Results report 63/168 = 37.5% missed at 12 months, but the Discussion later refers to 25.6%."

Response: We have corrected all numerical inconsistencies throughout the manuscript. The corrected data are:

Visit Timely Delayed Missed

12 months 100 (59.5%) 43 (25.6%) 25 (14.9%)

18 months 63 (37.5%) 44 (26.2%) 61 (36.3%)

Changes made:

• Lines 27-28: Abstract - Updated missed doses: "Missed doses were highest at 18 months (36.3%), followed by 12 months (14.9%)."

• Lines 28-29: Abstract - Updated timely vaccination: "Timely vaccination dropped sharply from 98.6% at birth to 52.4% at 9 months, and 37.5% at 18 months."

• Lines 280-296: Results - Fig 3 data - Updated all numbers for 12 and 18 months

• Lines 304-306: Figure 3 caption - Updated: "Timely vaccination declined sharply after birth, from 98.6% at birth to 52.4% at 9 months and 37.5% at 18 months. Missed doses were highest at 18 months (36.3%), followed by 12 months (14.9%)."

• Lines 329-331: Results - Fig 5 - Updated: "coverage declined to 85.1% (59.5% timely) at 12-month visit, and at the 18-month visit, coverage dropped further to 63.7% (37.5% timely)."

• Lines 293-298: Figure 5 caption - Updated coverage and timeliness data

• Lines 335-342: Discussion - Updated: "Missed vaccinations were highest at 18-month (36.3%)."

• Lines 417-420: Discussion - Updated: "Coverage dropped sharply after 9 months with only 85.1% coverage at 12 months and 63.7% at 18 months -- with missed doses reaching 36.3% at 18 months."

Comment 7: "Discussion is too long and somewhat speculative: Several paragraphs repeat the Results."

Response: We have substantially revised the Discussion section to eliminate redundancy, reduce speculation, and focus on interpretation and implications.

Changes made: Lines 397-459: Discussion section condensed; removed redundant restatement of visit-by-visit results; strengthened focus on implications

RESPONSE TO REVIEWER #2

Question 1: "Have the booster doses been taken into consideration?"

Response: Yes, booster doses were fully included in our assessment. The 18-month visit includes booster doses for DTaP-IPV-Hib, MMR-2, and Varicella-2.

Changes made:

• Lines 111-112: Methods - Study Population - Added: "completing both primary doses (birth till 12-month) and booster doses at 18-month"

• Lines 113-117: Methods - Study Population - Added complete vaccine list with booster doses at 18 months

• Lines 418-420: Discussion - Added: "The high missed dose rate at 18 months is particularly concerning as this visit includes critical booster doses needed to maintain immunity through early childhood."

Question 2: "An important chunk of delay also happens after 2 years. How has that been addressed?"

Response: We acknowledge this limitation and have addressed it in the Methods justification, Limitations, and Recommendations.

Changes made:

• Lines 118-120: Methods - Study Population - Added justification: "the age window (18-24 months) was selected to minimize the recall bias as immunization cards are still typically retained by caregivers during this period."

• Lines 501-506: Discussion - Limitations - Added: "This study focused exclusively on children aged 18-24 months, not capturing vaccination delays or missed opportunities occurring after 2 years of age, including booster doses scheduled at 4-6 years (DTaP-IPV, MMR) and later childhood vaccines."

• Line 509-510: Discussion - Limitations - Added: "Future research should employ mixed-methods approaches to capture multi-stakeholder perspectives."

Question 3: "Describe a post conflict Urban setting wrt Health care worker availability, duration since conflict, damage done etc."

Response: We have added a comprehensive description of the post-conflict urban setting in Baghdad.

Changes made: Lines 88-95: Methods - Study Design - Added: "Baghdad represents a post-conflict healthcare context due to decades of war and instability, experienced multiple conflicts since 1980, including Iran-Iraq war (1980-1988), the Gulf war (1990-1991), 2003 invasion and occupation, and the ISIS conflict (2014-2017). Although major active conflict subsided for more than two decades, the healthcare system continues to struggle with cumulative effects. Availability of healthcare worker is still a challenge with approximately 1.8 physicians per 1000 population, lower than numbers of 2003 [11, 12]. Many primary health care centres face resource challenges, and the cold-chain compromised by electricity shortages [12]."

Question 4: "Brief the Health care system in Urban Baghdad. Do PHCs also exist in urban area? If yes, what is the population it caters to?"

Response: We have added a detailed description of the healthcare system in urban Baghdad.

Changes made: Lines 97102: Methods - Study Design - Added: "Baghdad has a mix of public and private health care facilities. Primary Healthcare Centres (PHCs) are the primary public health facility, providing free of cost preventive and curative services, that include routine immunization. PHCs in urban Baghdad normally serve to populations of 15,000-50,000 residents in the neighbourhoods, as the first point of contact for maternal and child health services. These PHCs deliver EPI services, which make them strategically important for the evaluation of vaccination program performance in the urban setting [11, 13]."

Question 5: "What are the visits at 4 and 6 months for. Brief about the Immunisation schedule under the Iraq Ministry."

Response: We have added the complete Iraqi immunization schedule with vaccine names.

Changes made:

Lines 113-117: Methods - Study Population - Added complete vaccine list: "These seven essential immunization visits included: birth (1st week: BCG, OPV-0, HepB-1), 2 months (DTaP-HepB-Hib-1, OPV-1, PCV-1, Rotavirus-1), 4 months (DTaP-HepB-Hib-2, OPV-2, PCV-2, Rotavirus-2), 6 months (DTaP-HepB-Hib-3, OPV-3, PCV-3), 9 months (Measles-1), 12 months (MMR-1, Varicella-1), and 18 months (DTaP-IPV-Hib Booster, MMR-2, Varicella-2) [16, 17]."

Question 6: "Calculating Sample size by considering only the measles coverage might lead to miscalculations. Either take complete immunisation coverage or prevalence of delay."

Response: We acknowledge this limitation. At the time of study design, the most recently published immunization coverage estimate for Iraq was measles vaccination coverage (83.7%) [18], and complete immunization coverage data was not available. Our final sample of 168 exceeded the minimum requirement of 10 events per predictor variable (EPV ratio 22:1).

Changes made: No manuscript change made

Question 7: "Describe properly the multistage stratification that was applied?"

Response: We have added a detailed description of the multistage stratified purposive sampling technique.

Changes made: Lines 139-152: Methods - Sampling Method - Added detailed description of Stage 1 (PHC selection), Stage 2 (participant selection), Stage 3 (enrolment), and rationale.

Question 8: "How was missing data addressed?"

Response: Missing data handling was already described in the Methods section.

Changes made: No manuscript change needed - Already addressed in original manuscript at lines 224-228.

Question 9: "May remove or merge the rows with too less values [< 5]"

Response: We have merged small categories in Table 1.

Changes made:

• Lines 263: Table 1 - Mothers Age: Merged to "Between 30 years" (n=105, 62.5%)

• Lines 263: Table 1 - Fathers Age: Removed "Below 20 years" row (n=0)

• Line 263: Table 1 - Added footnote: "Categories with small numbers (<5) were merged where appropriate to ensure stable estimates."

Question 10: "Is the multiple visit delay inclusive of the single delays?"

Response: Single and multiple visit delays are mutually exclusive categories.

Changes made:

• Lines 268-269: Results - Added: "These categories are mutually exclusive."

• Lines 274-277: Figure 2 caption - Added: "Among these, 28 children (16.7%) were delayed at only one visit (single visit delay), and 126 children (75.0%) were delayed at two or more visits (multiple visit delay). These categories are mutually exclusive."

Question 11: "What was the cut off for missed vaccination & differentiate it well from the Delays?"

Response: We have added a clear distinction between "delayed" and "missed" vaccinations.

Changes made:

• Lines 179-182: Methods - Study Variables - Added: "A missed vaccination was defined as a vaccine that was not administered by the time of data collection (i.e., no record of vaccination on the card for a particular scheduled visit by 18-24 months of age). This is distinct from delayed vaccination, where the vaccine was administered but beyond the 28-day window."

• Lines 303-305: Figure 3 caption - Added definitions: "Valid (Timely) = vaccine administered within 28 days of scheduled date, Delayed = vaccine administered >28 days after scheduled date, Missed = no record of vaccination on card by 18-24 months of age."

Question 12: "Why haven't the adjusted Odds ratio for rest of the factors taken out... Needed a mention on the factors that were taken forward and the cut off used."

Response: We have added a comprehensive explanation of the model building process.

Changes made:

• Lines 220-224: Methods - Data Analysis - Added model building process with EPV calculation

• Lines 350-352: Results - Before Table 2 - Added: "Table 2 presents both unadjusted odds ratios (from bivariate analysis) and adjusted odds ratios from the final multivariate logistic regression model. Adjusted odds ratios are presented only for variables that were retained in the final model after backward elimination."

• Lines 364: Table 2 footnote - Added: "Adjusted ORs are shown only for variables retained in the final model after backward elimination. *Statistically significant at p < 0.05."

Question 13: "The perspective of multiple stakeholders in finding out the reasons for delay would provide better cues"

Response: We have added this as a limitation and recommendation for future research.

Changes made: Lines 505-509: Discussion - Limitations - Added: "The reasons for immunization delay were assessed only from caregiver perspectives. Inputs from healthcare providers, PHC administration, community health workers, and policymakers, can give a comprehensive outlook of systemic and structural barriers to timely vaccination. Future research should employ mixed-methods approaches to capture multi-stakeholder perspectives."

Question 14: "Try to write recommendations based on study findings"

Response: We have revised the recommendations section to be directly grounded in our specific study findings.

Changes made: Lines 468-481: Revised to be evidence-based with specific percentages linked to each recommendation.

Question 15: "Check the referencing type and maintain uniformity"

Response: We have carefully reviewed and standardized all references according to PLOS ONE requirements.

Changes made: References section reformatted to PLOS ONE style, missing DOI added.

RESPONSE TO ACADEMIC EDITOR

Requirement 1: "Please ensure that your manuscript meets PLOS ONE's style requirements, including those for file naming."

Response: We have reformatted the manuscript according to PLOS ONE's style templates and file naming.

Requirement 2: "In the online submission form, you indicated that 'The original data are available from the corresponding author upon reasonable request.' All PLOS journals now require all data underlying the findings described in their manuscript to be freely available..."

Response: We have revised the Data Availability Statement to ensure full compliance.

Changes made: Lines 541-545: Data Availability Statement - Revised to: "All data underlying the findings are fully available without restriction. The anonymized dataset supporting the findings of this study is available as Supporting Information (S4 File). The data have been anonymized to protect participant confidentiality."

Requirement 3: "We note you have included a table to which you do not refer in the text of your manuscript. Please ensure that you refer to Table 1 in your text..."

Response: We have added a reference to Table 1 in the Results section.

Changes made: Line 250: Results - Added: "The demographic characteristics of the study participants are presented in Table 1."

Requirement 4: "If the reviewer comments include a recommendation to cite specific previously published works, please review and evaluate these publications..."

Response: No specific papers were suggested by either reviewer.

CONCLUSION

We are grateful for the thorough and constructive reviews. We believe the revised manuscript is substantially improved, with clearer methodology, more cautious interpretation of findings, stronger acknowledgment of limitations, and more actionable, evidence-based recommendations. We have addressed all reviewer and editor concerns systematically and hope the revisions meet the standards for publication in PLOS ONE.

We look forward to your response and remain available for any additional clarifications.

Sincerely,

Dr. Syed Shaukat Ali Muttaqi Shah & Dr. Ale Zehra

Corresponding Authors

Attachment Submitted filename: Point by point response to reviewer comments.docx

# Editor report: Decision Letter 1

**DOI:** 10.1371/journal.pone.0359962.r003

**Authors:** Mohammed Badeso

22 Sep 2026

<p>Immunization Timeliness and Its Determinants Among Young Children in Baghdad, Iraq: A Cross-Sectional Study Using Documented Vaccination Cards

PONE-D-26-19652R1

Dear Dr. Shah,

We’re pleased to inform you that your manuscript has been judged scientifically suitable for publication and will be formally accepted for publication once it meets all outstanding technical requirements.

Within one week, you’ll receive an e-mail detailing the required amendments. When these have been addressed, you’ll receive a formal acceptance letter and your manuscript will be scheduled for publication.

An invoice will be generated when your article is formally accepted. Please note, if your institution has a publishing partnership with PLOS and your article meets the relevant criteria, all or part of your publication costs will be covered. Please make sure your user information is up-to-date by logging into Editorial Manager at Editorial Manager® and clicking the ‘Update My Information' link at the top of the page. For questions related to billing, please contact billing support.

If your institution or institutions have a press office, please notify them about your upcoming paper to help maximize its impact. If they’ll be preparing press materials, please inform our press team as soon as possible -- no later than 48 hours after receiving the formal acceptance. Your manuscript will remain under strict press embargo until 2 pm Eastern Time on the date of publication. For more information, please contact onepress@plos.org.

Kind regards,

Mohammed Hasen Badeso, Epidemiologist

Academic Editor

PLOS One

Additional Editor Comments (optional):

Reviewers' comments:

# Editor report: Acceptance letter

**DOI:** 10.1371/journal.pone.0359962.r004

**Authors:** Mohammed Badeso

PONE-D-26-19652R1

PLOS One

Dear Dr. Shah,

I'm pleased to inform you that your manuscript has been deemed suitable for publication in PLOS One. Congratulations! Your manuscript is now being handed over to our production team.

At this stage, our production department will prepare your paper for publication. This includes ensuring the following:

* All references, tables, and figures are properly cited

* All relevant supporting information is included in the manuscript submission,

* There are no issues that prevent the paper from being properly typeset

You will receive further instructions from the production team, including instructions on how to review your proof when it is ready. Please keep in mind that we are working through a large volume of accepted articles, so please give us a few days to review your paper and let you know the next and final steps.

Lastly, if your institution or institutions have a press office, please let them know about your upcoming paper now to help maximize its impact. If they'll be preparing press materials, please inform our press team within the next 48 hours. Your manuscript will remain under strict press embargo until 2 pm Eastern Time on the date of publication. For more information, please contact onepress@plos.org.

You will receive an invoice from PLOS for your publication fee after your manuscript has reached the completed accept phase. If you receive an email requesting payment before acceptance or for any other service, this may be a phishing scheme. Learn how to identify phishing emails and protect your accounts at https://explore.plos.org/phishing.

If we can help with anything else, please email us at customercare@plos.org.

Thank you for submitting your work to PLOS One and supporting open access.

Kind regards,

PLOS One Editorial Office Staff

on behalf of

Mr Mohammed Hasen Badeso

Academic Editor

PLOS One
